# Supplementary material for: Kinetics of the ancestral carbon metabolism pathways in deep-branching bacteria and archaea
Source: Commun Chem. 2021 Oct 22;4:149. doi: 10.1038/s42004-021-00585-0 (PMC9814661; doi:10.1038/s42004-021-00585-0)
Supplement: Supplementary file 2 — Supplementary Information [file 42004_2021_585_MOESM2_ESM.pdf]

Supplementary Information for

Kinetics of the ancestral carbon metabolism pathways in deep-branching bacteria and archaea

Tomonari Sumi[1,2] and Kouji Harada[3,4]

[1] Research Institute for Interdisciplinary Science, Okayama University, 3-1-1 Tsushima-Naka, Kita-ku, Okayama 700-8530, Japan
[2] Department of Chemistry, Faculty of Science, Okayama University, 3-1-1 Tsushima-Naka, Kita-ku, Okayama 700-8530, Japan
[3] Department of Computer Science and Engineering, Toyohashi University of Technology, Tempaku-cho, Toyohashi 441-8580, Japan
[4] Center for IT-Based Education, Toyohashi University of Technology, Tempaku-cho, Toyohashi, Aichi, 441-8580, Japan

Kinetic network model for carbon metabolism related to the rTCA cycle of *T. takaii*

The kinetic network model is mathematically described by using the ordinary differential equations. The metabolites shown in Fig. A1 are included as the reactant or product in enzymatic reactions and biomass synthesis reactions described below (Eqs. B1–B18). The ordinary differential equations are described using the reaction fluxes shown in Fig. A1 as follows.

$d[\mathrm{CIT}]/dt = J_{cs} - J_{ac},$ (1)

$d[\mathrm{ICIT}]/dt = J_{ac} - J_{icdh},$ (2)

$d[\mathrm{AKG}]/dt = J_{icdh} + J_{akgsyn} - J_{growth}(\mathrm{AKG}),$ (3)

$d[\mathrm{SCOA}]/dt = -J_{akgsyn} - J_{scas},$ (4)

$d[\mathrm{SUC}]/dt = J_{scas} - J_{sdh},$ (5)

$d[\mathrm{FUM}]/dt = J_{sdh} - J_{fuma},$ (6)

$d[\mathrm{MAL}]/dt = J_{fuma} - J_{mdh} - J_{me},$ (7)

$d[\mathrm{OAA}]/dt = J_{mdh} + J_{pyc} - J_{cs} - J_{ppck} - J_{growth}(\mathrm{OAA}),$ (8)

$d[\mathrm{PYR}]/dt = J_{me} + J_{pys} - J_{pyc} - J_{pps} - J_{pyk} - J_{growth}(\mathrm{PYR}),$ (9)

$d[\mathrm{PEP}]/dt = J_{pps} + J_{pyk} + J_{ppck} - J_{growth}(\mathrm{PEP}),$ (10)

$d[\mathrm{ACOA}]/dt = -J_{pys} - J_{cs} - J_{growth}(\mathrm{ACOA}),$ (11)

$d[\mathrm{NAD}]/dt = J_{fdx\_nad} - J_{mdh},$ (12)

$d[\mathrm{NADH}]/dt = J_{mdh} - J_{fdx\_nad},$ (13)

$d[\mathrm{NADP}]/dt = J_{fdx\_nadp} - J_{icdh} - J_{me},$ (14)

$d[\mathrm{NADPH}]/dt = J_{icdh} + J_{me} - J_{fdx\_nadp},$ (15)

$$d[\text{AMP}]/dt = J_{pyk} - J_{adk}. \quad (16)$$

The steady state concentrations and fluxes are determined by Eqs. 1–16 under fixed concentrations of the chemical species listed in Table A1.

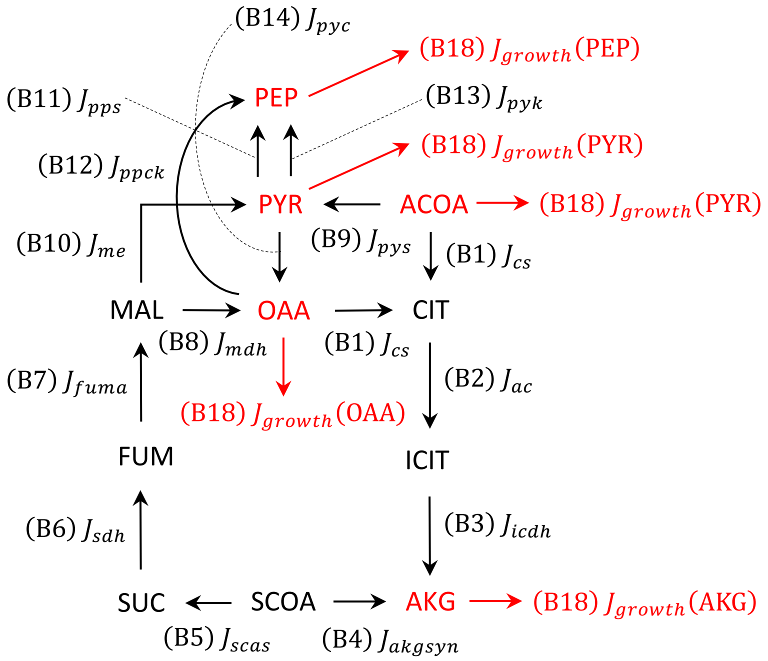

Figure A1. Enzymatic reactions and biomass synthesis included in the kinetic network model for carbon metabolism related to the rTCA cycle of *T. takaii*. The black and red solid arrows indicate the positive direction of flux J for enzymatic reactions defined by Eq. B1–B14 and biomass synthesis reactions by Eq. B18, respectively.

General aspects on kinetic modeling of enzymatic reactions

The kinetic modeling of enzymatic reaction is basically conducted according to that by Beard and co-workers [1]. The overall concentration of all species of reactant is employed to construct the kinetic model of enzymatic reactions. For example, $[\text{CO}_2]$ denotes the overall concentration given by

$$[\text{CO}_2] = [\text{CO}_3^{2-}] + [\text{HCO}_3^-] + [\text{H}_2\text{CO}_3],$$

which is calculated using the binding polynomial $P_{\text{CO}_3^{2-}}$ as [2]

$$[\text{CO}_2] = P_{\text{CO}_3^{2-}}[\text{CO}_3^{2-}],$$

where

$$P_{\text{CO}_3^{2-}} = 1 + \frac{10^{-\text{pH}}}{K_{\text{H1}}} + \frac{10^{-2\text{pH}}}{K_{\text{H1}}K_{\text{H2}}}. \quad (\text{A1})$$

Here, pH is defined on the basis of the activity of free hydrogen ion, a_{H^+}, rather than its concentration $[\text{H}^+]$. Thus, $\text{pH} = -\log_{10}(a_{\text{H}^+}) = -\log_{10}([\text{H}^+]\cdot\gamma_{\text{H}^+})$, where γ_{H^+} is the activity coefficient of hydrogen ion. K_{H1} is the dissociation constant of H^+, and given by $K_{\text{H1}} = 10^{-\text{p}K_{\text{H1}}}$. The general form of the binding polynomial is given by

$$P_j = 1 + \frac{10^{-\text{pH}}}{K_{\text{H1}}} + \frac{10^{-2\text{pH}}}{K_{\text{H1}}K_{\text{H2}}} + \frac{[\text{Mg}^{2+}]}{K_{\text{Mg1}}} + \frac{10^{-\text{pH}}[\text{Mg}^{2+}]}{K_{\text{H1}}K_{\text{Mg1}}} + \frac{[\text{Mg}^{2+}]^2}{K_{\text{Mg1}}K_{\text{Mg2}}} + \cdots. \quad \text{(A2)}$$

The details of the binding polynomial are presented in the database provided by Beard and co-workers [2].

The standard-state reaction Gibbs free energy for reference reactions is used to calculate an apparent equilibrium constant for the overall concentrations of all species of reference reactant. The standard state of the reference reactions is at temperature T_s = 298.15 K and ionic strength I_s= 0 M. It has been proposed that the effects of temperature and ionic strength on the reaction Gibbs free energy for the reference reactions are approximately calculated over the temperature range between 273.15 K and 313.15 K using the van't Hoff relationship and the extended Debye-Hückel theory [2-4].

$$\Delta_r G^0(T,I) = \frac{T}{T_s}\Delta_r G^0(T_s,I_s) + \left(1-\frac{T}{T_s}\right)\Delta_r H^0(T_s,I_s) - RT\alpha(T)\frac{I^{1/2}}{1+BI^{1/2}}\sum_{i=1}^{N} \nu_i z_i^2, \quad \text{(A3)}$$

$$\alpha(T) = 1.10708 - (1.54508 \times 10^{-3})T + (5.955884 \times 10^{-6})T^2, \quad \text{(A4)}$$

where $\Delta_r H^0$ is assumed to be constant over the temperature region. Here, R is the gas constant, N is the total number of reference species, ν_i is the stoichiometric coefficient, which is negative for the substrates and positive for the products, I is the ionic strength in Molar (M) units, and B is an empirical constant taken to be 1.6 $M^{-1/2}$. $\alpha(T)$ in Eq. A4 is an empirical function that varies with temperature. The activity coefficient of an ionic species j with the valence z_j is also provided by the extended Debye-Hückel theory [2-4],

$$\ln\gamma_{z_j} = -\frac{\alpha(T)z_j^2 I^{1/2}}{1+BI^{1/2}}. \quad \text{(A5)}$$

Thus, the activity of an ionic species j is calculated via $a_j = \gamma_{z_j}[j]$ with the concentration $[j]$. The equilibrium constant for the reference reaction is defined by

$$K_{eq}^0 = \exp\left(\frac{-\Delta_r G^0(T,I)}{RT}\right). \quad \text{(A6)}$$

This should be converted into the apparent equilibrium constant for the associated biochemical reaction K_{eq}^{tot} which is useful to construct the kinetic model of enzymatic reactions.

$$K_{eq}^{tot} = \frac{K_{eq}^0}{(a_{\text{H}^+})^{\nu_{\text{H}^+}}(a_{\text{H}_2\text{O}})^{\nu_{\text{H}_2\text{O}}}}\frac{\prod_{i=1}^{N'} P_i^{\nu_i}}{\prod_{i=1}^{N'} \gamma_{z_i}^{\nu_i}}, \quad \text{(A7)}$$

where N' is the total number of the reference species except for H^+ and H_2O. By using the apparent equilibrium constant K_{eq}^{tot} provided by Eq. A7, the flux of the kinetic model is consistent with the reaction Gibbs free energy,

$$\Delta_r G(T,I) = \Delta_r G^0(T,I) + RT\ln\left[\prod_{i=1}^{N} a_i^{\nu_i}\right]. \quad \text{(A8)}$$

It is noted that N in Eq. A8 is the total number of all species in the reference reaction. Here, to characterize the exergonicity of enzymatic reaction, the reaction Gibbs free energy with the apparent equilibrium constant of Eq. A7 is define as

$$\Delta_r G^{tot}(T,I) = -RT\ln K_{eq}^{tot}, \quad \text{(A9)}$$

which is evaluated for each enzymatic reaction and summarized in Table S2. It is noted that the apparent reaction Gibbs free energy $\Delta_r G^{tot}(T, I)$ by Eq. A9 is introduced to characterize the exergonicity of each enzymatic reaction. As seen in Table S3, even if the enzymatic reaction is significantly exergonic with huge negative value of $\Delta_r G_i^{tot}$, it can be reversal by high reactant and low product concentrations of the reversed reaction, as shown by Eq. A8 of the reaction Gibbs free energy. The apparent equilibrium constant Eq. A7 is used in the kinetic model of enzymatic reactions given below, so that the rate of enzymatic reactions is determined by the overall concentrations of reactants and products obtained from the binding polynomial Eq. A2. The direction of reaction determined by the kinetic models of (enzymatic) reactions (Eqs. B1–B17) depends on the concentrations of reactants and products and is consistent with the reaction Gibbs free energy given by Eq. A8. All the results discussed by the text and SI are based on the steady state concentrations and fluxes that are determined by the kinetic network model simulations based on the ordinary differential equations (1)–(16).

Kinetic model of enzymatic reactions for TCA cycle

The data of $\Delta_r H^0(T_s, I_s)$ is not available for most of enzymatic reactions on TCA cycle [2,5], thus temperature effects on kinetic properties of rTCA cycle cannot be properly examined. Therefore, our calculations are commonly performed at T = 298.15 K. The parameters in the kinetic model of enzymatic reactions used for simulations of the physiological condition (T = 298.15 K, pH = 7.5, I = 0.18 M, [Mg^{2+}] = 0.8 mM, [K^+] = 140 mM) are provided below.

TCA cycle reactions

1. Citrate synthase (EC:2.3.3.1)

The reference reaction is

$OAA^{2-} + ACOA^0 + H_2O^0 = CIT^{3-} + COA^- + 2\ H^+$.

The standard-state reaction Gibbs free energy for the reference reaction (T_s = 298.15 K, I_s = 0 M) is calculated using the database, [2]

$$\Delta_r G^0_{cs}(T_s, I_s) = \Delta_f G^0_{\mathrm{CIT^{3-}}} + \Delta_f G^0_{\mathrm{COA^-}} + 2\Delta_f G^0_{\mathrm{H^+}} - \Delta_f G^0_{\mathrm{OAA^{2-}}} - \Delta_f G^0_{\mathrm{ACOA^0}} - \Delta_f G^0_{\mathrm{H_2O^0}} = 60.32\ \mathrm{kJ/mol}.$$

Using the reaction Gibbs free energy for the reference reaction (Eq. A3) at T = 298.15 K, pH = 7.5, I = 0.18 M, the apparent equilibrium constant (Eq. A7) is calculated as

$$K_{eq}^{tot} = 2.294566\ \times 10^9\ \mathrm{kJ/mol},$$

which is applied to the rate equation of Eq. B1. The apparent reaction Gibbs energy (Eq. A9) is obtained as

$$\Delta_r G_{cs}^{tot}(T, I) = -53.43\ \ \mathrm{kJ/mol}.$$

The biochemical reaction for the overall concentration of all species of reactant is

$OAA + ACOA + H_2O = COA + CIT + 2\ H^+$.

The reactant concentrations are denoted as [OAA] = substrateA, [ACOA] = substrateB, [COA] = productP, and [CIT] = productQ in the following enzymatic rate equation [6]:

J_{cs} = V_f/((KeA*KmB)*(1+[H^+]/KiH))*(substrateA*substrateB -productP*productQ /Keq) /[(1+KmA/(KeA*KmB)*(substrateB)+KmQ/(KeQ*KmP)*(productP))+(substrateA/KeA+KmQ/(KeA*KmP *KeQ)*substrateA*productP+KmA/(KeA^2*KmB*Keq))*(1+(ATP/P_ATP)/KiATP+(ADP/P_ADP)/KiADP +(SCOA/P_SCOA)/KiSCOA)+(1/(KeA*KmB)-KmQ*Keq/(KeQ^2*KmP))*substrateA*substrateB+(1/(Km P*KeQ)-KmA/(KeA^2*KmB*Keq))*productP*productQ+KmQ/(KeA*KeB*KmP*KeQ)*substrateA*substr ateB*productP+KmA/(KeA*KmB*KeP*KeQ)*substrateB*productP*productQ+productQ/KeQ+KmQ*Keq/ (KeQ^2*KmP)*substrateA*substrateB+KmA/(KeA*KmB*KeQ)*substrateB*productQ], (B1)

where P_ATP, P_ADP, and P_SCOA are the binding polynomial [2] given by Eq. A2. The parameters in Eq B1 are taken from the literature [6].

KmA	1.3700E-06	M
KmB	1.4400E-05	M
KmP0	1.5000E-07	M
KmQ	4.6300E-03	M
KeA	9.1000E-07	M
KeB	2.9700E-05	M
KeQ	3.9300E-03	M
KiATP	3.7300E-05	M
kiADP	1.3540E-04	M
KiSCOA	7.4100E-05	M
KiH	5.5000E-08	M
KmP	1.5000E-06	M (This work)
KeP	7.8091E+00	From Eq. 11 of Ref. [6]

2. Aconitate (EC: 4.2.1.3)

The reference reaction is

$CIT^{3-} = ICIT^{3-}$.

The standard-state reaction Gibbs free energy for the reference reaction (T_s = 298.15 K, I_s = 0 M) is calculated using the database, [2]

$\Delta_r G^0_{ac}(T_s, I_s) = \Delta_f G^0_{\mathrm{ICIT^{3-}}} - \Delta_f G^0_{\mathrm{CIT^{3-}}} = 5.76$ kJ/mol.

Using the reaction Gibbs free energy for the reference reaction (Eq. A3) at T = 298.15 K, pH = 7.5, I = 0.18 M, the apparent equilibrium constant (Eq. A7) is calculated as

$K^{tot}_{eq} = 3.158767 \times 10^{-2}$,

which is applied to the rate equation of Eq. B2. The apparent reaction Gibbs energy (Eq. A9) is obtained as

$\Delta_r G^{tot}_{ac}(T, I) = 8.56$ kJ/mol.

The biochemical reaction for the overall concentration of all species of reactant is

CIT = ICIT.

The reactant concentrations are denoted as [CIT] = substrateA and [ICIT] = productP in the following enzymatic rate equation [1]:

J_{ac} = Vf*(substrateA-productP/Keq)/(KmA+substrateA+KmA*productP/KmP). (B2)

The parameters in Eq B2 are taken from the literatures [1].

KmA	1.1610E-03	M
KmP	4.3400E-04	M

3. Isocitrate dehydrogenase (EC:1.1.1.42)

The reference reaction is

$NADP^{3-} + ICIT^{3-} + H_2O^0 = AKG^{2-} + NADPH^{4-} + CO_3^{2-} + 2\ H^+$.

The standard-state reaction Gibbs free energy for the reference reaction (T_s = 298.15 K, I_s = 0 M) is calculated using the database, [2]

$$\Delta_r G^0_{icdh}(T_s, I_s) = \Delta_f G^0_{\mathrm{AKG^{2-}}} + \Delta_f G^0_{\mathrm{NADPH^{4-}}} + \Delta_f G^0_{\mathrm{CO_3^{2-}}} + 2\Delta_f G^0_{\mathrm{H^+}} - \Delta_f G^0_{\mathrm{ICIT^{3-}}} - \Delta_f G^0_{\mathrm{NADP^{3-}}} - \Delta_f G^0_{\mathrm{H_2O}} = 97.06\ \mathrm{kJ/mol}.$$

Using the reaction Gibbs free energy for the reference reaction (Eq. A3) at T = 298.15 K, pH = 7.5, I = 0.18 M, the apparent equilibrium constant (Eq. A7) is calculated as

$$K_{eq}^{tot} = 2.958463 \times 10^5,$$

which is applied to the rate equation of Eq. B3. The apparent reaction Gibbs energy (Eq. A9) is obtained as

$$\Delta_r G_{icdh}^{tot}(T, I) = -0.12\ \mathrm{kJ/mol}.$$

The biochemical reaction for the overall concentration of all species of reactant is

NADP + ICIT + H_2O = AKG + NADPH + CO_2(tot) + 2 H^+.

The reactant concentrations are denoted as [NADP] = substrateA, [ICIT] = substrateB, [AKG] = productP, [NADPH] = productQ, and [CO_2(tot)] = productR in the following enzymatic rate equation [7]:

J_{icdh} =

Vf/(1+[H^+]/KiH)/Kia*substrateA*(γ_1*β_{MgIcit}*substrateB/KmB)^3/(1+substrateA/Kia+KmA/Kia*(γ_1*β_{MgIcit}*substrateB/KmB)^3+productQ/Kiq+(substrateA/Kia)*(γ_1*β_{MgIcit}*substrateB/KmB)^3+(productQ/Kiq)*KmA/Kia*(γ_1*β_{MgIcit}*substrateB/KmB)^3)-Vf/(1+[H^+]/KiH)/(Kia*KmB^3)*γ_1^3*(substrateB*β_{MgIcit})^2*productP*productQ*productR/Keq/(1+substrateA/Kia+KmA/Kia*(γ_1*β_{MgIcit}*substrateB/KmB)^3+productQ/Kiq+(substrateA/Kia)*(γ_1*β_{MgIcit}*substrateB/KmB)^3+(productQ/Kiq)*KmA/Kia*(γ_1*β_{MgIcit}*substrateB/KmB)^3),

(B3)

where [H^+] = 10^{-pH}, γ_1 is the activity coefficient of monovalent ion provided by Eq. A5, and β_{MgIcit} = ([Mg^{2+}]/10^{-pKMg1})$P_{ICIT^{3-}}$ [2].

The parameters in Eq B3 are taken from the literatures for NAD-dependent isocitrate dehydrogenase [7].

KmA	5.0330E-04	M

KmB	1.4890E-04	M
Kia	7.7600E-05	M
Kiq	4.7500E-06	M
KiH	1.1000E-07	M

4. 2-oxoglutarate synthase (EC: C1.2.7.3) (or 2-oxoglutarate: ferredoxin oxidoreductase)

The reference reaction is

2 Fdx_red^{+} + $SCOA^{-}$ + CO_3^{2-} + 2 H^{+} = 2 Fdx_ox^{2+} + COA^{-} + AKG^{2-} + H_2O^0.

The standard-state reaction Gibbs free energy for the reference reaction (T_s = 298.15 K, I_s = 0 M) is calculated using the database, [2]

$\Delta_r G^0_{akgsyn}(T_s, I_s) = 2\Delta_f G^0_{\mathrm{Fdx\_ox^{2+}}} + \Delta_f G^0_{\mathrm{COA^-}} + \Delta_f G^0_{\mathrm{AKG^{2-}}} + \Delta_f G^0_{\mathrm{H_2O^0}} - 2\Delta_f G^0_{\mathrm{Fdx\_red^+}} - \Delta_f G^0_{\mathrm{SCOA^-}} - \Delta_f G^0_{\mathrm{CO_3^{2-}}} - 2\Delta_f G^0_{\mathrm{H^+}} = -64.76\ \mathrm{kJ/mol}$. Here, the standard-state Gibbs free energy change of ferredoxin (Fdx) upon the reduction is experimentally determined as $\Delta_r G^0_{\mathrm{Fdx\_red}} \equiv \Delta_f G^0_{\mathrm{Fdx\_red^+}} - \Delta_f G^0_{\mathrm{Fdx\_ox^{2+}}} = 36.7\ \mathrm{kJ/mol}$ [8]. Using the reaction Gibbs free energy for the reference reaction (Eq. A3) at T = 298.15 K, pH = 7.5, I = 0.18 M, the apparent equilibrium constant (Eq. A7) is calculated as

$K_{eq}^{tot} = 1.680798 \times 10^{-6}$,

which is applied to the rate equation of Eq. B4. The apparent reaction Gibbs energy (Eq. A9) is obtained as

$\Delta_r G^{tot}_{akgsyn}(T, I) = 32.96\ \mathrm{kJ/mol}$.

The biochemical reaction for the overall concentration of all species of reactant is

2 Fdx_red + SCOA + CO_2(tot) + 2 H^{+} = 2 Fdx_ox + COA + AKG + H_2O.

The reactant concentrations are denoted as [Fdx_red] = substrateA, [SCOA] = substrateB, [CO_2(tot)] = substrateC, [Fdx_ox] = productP, [COA] = productQ, and [AKG] = productR in the following enzymatic rate equation:

J_{akgsyn} = Vf*(substrateA*substrateA*substrateB*substrateC-productP*productP*productQ*productR/Keq). (B4)

4'. 2-oxoglutarate dehydrogenase (EC: 1.2.1.ak)

The reference reaction is

AKG^{2-} + COA^{-} + NAD^{-} + H_2O^0 = CO_3^{2-} + $SCOA^{-}$ + $NADH^{2-}$ + H^{+}.

The standard-state reaction Gibbs free energy for the reference reaction (T_s = 298.15 K, I_s = 0 M) is calculated using the database, [2]

$\Delta_r G^0_{adh}(T_s, I_s) = \Delta_f G^0_{\mathrm{CO_3^{2-}}} + \Delta_f G^0_{\mathrm{SCOA^-}} + \Delta_f G^0_{\mathrm{NADH^{2-}}} + \Delta_f G^0_{\mathrm{H^+}} - \Delta_f G^0_{\mathrm{AKG^{2-}}} - \Delta_f G^0_{\mathrm{COA^-}} - \Delta_f G^0_{\mathrm{NAD^-}} - \Delta_f G^0_{\mathrm{H_2O^0}} = 15.27\ \mathrm{kJ/mol}$.

Using the reaction Gibbs free energy for the reference reaction (Eq. A3) at T = 298.15 K, pH = 7.5, I = 0.18 M, the apparent equilibrium constant (Eq. A7) is calculated as

$K_{eq}^{tot} = 1.377646 \times 10^{9}$,

which is applied to the rate equation of Eq. B4'. The apparent reaction Gibbs energy (Eq. A9) is obtained as $\Delta_r G_{adh}^{tot}(T, I) = -52.16$ kJ/mol.

The biochemical reaction for the overall concentration of all species of reactant is

AKG + COA + H_2O + NAD = SCOA + CO_2(tot) + NADH + H^+.

The reactant concentrations are denoted as [AKG] = substrateA, [COA] = substrateB, [NAD] = substrateC, [CO_2(tot)] = productP, [SCOA] = productQ, and [NADH] = productR in the following enzymatic rate equation [9]:

J_{adh} =

Vf*substrateA*substrateB*substrateC*((1+Bmg*[Mg2+]/(Amg*KaMg)+Bmg*[Mg2+]^2/(Amg*KaMg)^2)/(1+[Mg2+]/(Amg*KaMg)+[Mg2+]^2/(Amg*KaMg)^2))/(substrateA*substrateB*substrateC+KmC/Kir*substrateA*substrateB*productR+KmC*substrateA*substrateB+KmB/Kiq*substrateA*substrateC*productQ+KmB*substrateA*substrateC+KmB*Kir/(Kiq*Kir)*substrateA*productQ*productR+Kic*KmB/Kiq*substrateA*productQ+KmA*substrateB*substrateC*((1+[ATP]/KiATP)*(1+[ADP]/KaADP)*(1+[Mg2+]/KaMg+[Mg2+]^2/(KaMg^2))*(10^-KaH/[H+])/((1+[ATP]/(Aatp*KiATP))*(1+[ADP]/(Aadp*KaADP))*(1+[Mg2+]/(Amg*KaMg)+[Mg2+]^2/(Amg*KaMg)^2)))+Kia*KmC/Kir*substrateB*productR+KmB*Kic*Kia/(Kiq*Kir)*productQ*productR)-Vf*productP*productQ*productR/Keq*((1+Bmg*[Mg2+]/(Amg*KaMg)+Bmg*[Mg2+]^2/(Amg*KaMg)^2)/(1+[Mg2+]/(Amg*KaMg)+[Mg2+]^2/(Amg*KaMg)^2))/(substrateA*substrateB*substrateC+KmC/Kir*substrateA*substrateB*productR+KmC*substrateA*substrateB+KmB/Kiq*substrateA*substrateC*productQ+KmB*substrateA*substrateC+KmB*Kir/(Kiq*Kir)*substrateA*productQ*productR+Kic*KmB/Kiq*substrateA*productQ+KmA*substrateB*substrateC*((1+[ATP]/KiATP)*(1+[ADP]/KaADP)*(1+[Mg2+]/KaMg+[Mg2+]^2/(KaMg^2))*(10^-KaH/[H+])/((1+[ATP]/(Aatp*KiATP))*(1+[ADP]/(Aadp*KaADP))*(1+Mg/(Amg*KaMg)+[Mg2+]^2/(Amg*KaMg)^2)))+Kia*KmC/Kir*substrateB*productR+KmB*Kic*Kia/(Kiq*Kir)*productQ*productR). (B4')

The parameters in Eq B4' are taken from the literature [9].

KmA	2.7300E-04	M
KmB	6.9600E-06	M
KmC	9.8600E-05	M
Kia	7.5900E-02	M
Kir	2.4000E-03	M
Kic	1.1200E-04	M
Kiq	2.1800E-04	M
KaH	6.1100E+00	
KiATP	1.0600E-04	M
KaADP	3.0500E-04	M
kaMg	1.9490E-05	M

Aatp	6.6940E+00	
Aadp	1.7300E-01	
Amg	1.0000E+00	
Bmg	4.2220E+00	

5. Succinyl-CoA synthetase (EC: 6.2.1.5)

The reference reaction is

$ADP^{3-} + SCOA^{-} + Pi^{2-} = COA^{-} + SUC^{2-} + ATP^{4-} + H^{+}$.

The standard-state reaction Gibbs free energy for the reference reaction (T_s = 298.15 K, I_s = 0 M) is calculated using the database, [2]

$\Delta_r G^0_{scas}(T_s, I_s) = \Delta_f G^0_{\mathrm{COA^-}} + \Delta_f G^0_{\mathrm{SUC^{2-}}} + \Delta_f G^0_{\mathrm{ATP^{4-}}} + \Delta_f G^0_{\mathrm{H^+}} - \Delta_f G^0_{\mathrm{ADP^{3-}}} - \Delta_f G^0_{\mathrm{SCOA^-}} - \Delta_f G^0_{\mathrm{Pi^{2-}}} = 56.56$ kJ/mol.

Using the reaction Gibbs free energy for the reference reaction (Eq. A3) at T = 298.15 K, pH = 7.5, I = 0.18 M, the apparent equilibrium constant (Eq. A7) is calculated as

$K^{tot}_{eq} = 7.154614$,

which is applied to the rate equation of Eq. B5. The apparent reaction Gibbs energy (Eq. A9) is obtained as

$\Delta_r G^{tot}_{scas}(T, I) = -4.88$ kJ/mol.

The biochemical reaction for the overall concentration of all species of reactant is

$ADP + SCOA + Pi = COA + SUC + ATP + H^{+}$.

The reactant concentrations are denoted as [ADP] = substrateA, [SCOA] = substrateB, [Pi] = substrateC, [COA] = productP, [SUC] = productQ, and [ATP] = productR in the following enzymatic rate equation [1]:

J_{scas} =

Vf*(substrateA*substrateB*substrateC-productP*productQ*productR/Keq)/(Kia*Kib*KmC+Kib*KmC*substrateA+Kia*KmB*substrateC+KmC*substrateA*substrateB+KmB*substrateA*substrateC+KmA*substrateB*substrateC+substrateA*substrateB*substrateC+Vf_pVr_pKeq*Kir*KmQ*productP+Vf_pVr_pKeq*Kiq*KmP*productR+Vf_pVr_pKeq*KmR*productP*productQ+Vf_pVr_pKeq*KmQ*productP*productR+Vf_pVr_pKeq*KmP*productQ*productR+Vf_pVr_pKeq*productP*productQ*productR+Vf_pVr_pKeq*KmQ*Kir/Kia*substrateA*productP+Kia*KmB/Kir*substrateC*productR+Vf_pVr_pKeq*KmQ*Kir/(Kia*Kib)*substrateA*substrateB*productQ+KmA/Kir*substrateB*substrateC*productR+Vf_pVr_pKeq*KmR/Kia*substrateA*productP*productQ+Kia*KmB/(Kiq*Kir)*substrateC*productQ*productR+Vf_pVr_pKeq*Kir*KmQ/(Kia*Kib*Kic)*substrateA*substrateB*substrateC*productP+Vf_pVr_pKeq*Kip*KmR/(Kia*Kib*Kic)*substrateA*substrateB*substrateC*productQ+Vf_pVr_pKeq*KmR/(Kia*Kib)*substrateA*substrateB*productP*productQ+KmA/(Kiq*Kir)*substrateB*substrateC*productQ*productR+KmA*Kic/(Kip*Kiq*Kir)*substrateB*productP*productQ*productR+Kia*KmB/(Kip*Kiq*Kir)*substrateC*productP*productQ*productR+Vf_pVr_pKeq*KmR/(Kia*Kib*Kic)*substrateA*substrateB*substrateC*productP*productQ+KmA/(Kip*Kiq*Kir)*substrateB*substrateC*productP*productQ*productR), (B5)

where a Haldane relation provides

Vf_pVr_pKeq = Vf/Vr/Keq = Kia*Kib*KmC/(KmP*Kiq*Kir) [10]. The parameters in Eq B5 are taken from the literature [1].

KmA	1.6000E-05	M
KmB	5.5000E-05	M
KmC	6.6000E-04	M
KmP	2.0000E-05	M
KmQ	8.8000E-04	M
KmR	1.1100E-05	M
Kia	5.5000E-06	M
Kib	1.0000E-04	M
Kic	2.0000E-03	M
Kip	2.0000E-05	M
Kiq	3.0000E-03	M
Kir	1.1100E-05	M

6. Succinate dehydrogenase (EC: 1.3.5.1)

The reference reaction is

$SUC^{2-} + COQ^{0} = QH_2^{0} + FUM^{2-}$.

The standard-state reaction Gibbs free energy for the reference reaction (T_s = 298.15 K, I_s = 0 M) is calculated using the database, [2]

$$\Delta_r G^0_{sdh}(T_s, I_s) = \Delta_f G^0_{\mathrm{QH}_2^0} + \Delta_f G^0_{\mathrm{FUM}^{2-}} - \Delta_f G^0_{\mathrm{SUC}^{2-}} - \Delta_f G^0_{\mathrm{COQ}^0} = -3.1 \text{ kJ/mol.}$$

Using the reaction Gibbs free energy for the reference reaction (Eq. A3) at T = 298.15 K, pH = 7.5, I = 0.18 M, the apparent equilibrium constant (Eq. A7) is calculated as

$$K_{eq}^{tot} = 2.493936,$$

which is applied to the rate equation of Eq. B6. The apparent reaction Gibbs energy (Eq. A9) is obtained as

$$\Delta_r G^{tot}_{sdh}(T, I) = -2.2653 \text{ kJ/mol.}$$

The biochemical reaction for the overall concentration of all species of reactant is

$SUC + COQ = FUM + QH_2$.

The reactant concentrations are denoted as [SUC] = substrateA, [COQ] = substrateB, [QH_2] = productP, and [FUM] = productQ in the following enzymatic rate equation [1]:

J_{sdh} =

Vf*(substrateA*substrateB-productP*productQ/Keq)/(Kia*KmB*(1+[OAA]/KiOAA+substrateA/KaSUC+productQ/KaFUM)/(1+substrateA/KaSUC+productQ/KaFUM)+KmB*substrateA+KmA*substrateB*(1+[OA

A]/KiOAA+substrateA/KaSUC+productQ/KaFUM)/(1+substrateA/KaSUC+productQ/KaFUM)+Vf_pVr_pKeq*KmQ*productP*(1+[OAA]/KiOAA+substrateA/KaSUC+productQ/KaFUM)/(1+substrateA/KaSUC+productQ/KaFUM)+Vf_pVr_pKeq*KmP*productQ+substrateA*substrateB+Vf_pVr_pKeq*KmQ/Kia*substrateA*productP+KmA/Kiq*substrateB*productQ+Vf_pVr_pKeq*productP*productQ), (B6)

where a Haldane relation provides

Vf_pVr_pKeq=Vf/Vr/Keq=Kia*KmB/(KmP*Kiq) [10]. The parameters in Eq B6 are taken from the literature [1].

KmA	4.6700E-04	M
KmB	4.8000E-04	M
KmP	2.4500E-06	M
KmQ	1.2000E-03	M
Kia	1.2000E-04	M
Kiq	1.2750E-03	M
KiOAA	1.5000E-06	M
KaSUC	4.5000E-04	M
KaFUM	3.7500E-04	M

7. Fumarate hydratase (EC: 4.2.1.2) (or Fumarase)

The reference reaction is

$FUM^{2-} + H_2O^0 = MAL^{2-}$.

The standard-state reaction Gibbs free energy for the reference reaction (T_s = 298.15 K, I_s = 0 M) is calculated using the database, [2]

$\Delta_r G^0_{fuma}(T_s, I_s) = \Delta_f G^0_{\mathrm{MAL^{2-}}} - \Delta_f G^0_{\mathrm{FUM^{2-}}} - \Delta_f G^0_{\mathrm{H_2O}} = -3.37$ kJ/mol.

Using the reaction Gibbs free energy for the reference reaction (Eq. A3) at T = 298.15 K, pH = 7.5, I = 0.18 M, the apparent equilibrium constant (Eq. A7) is calculated as

$K^{tot}_{eq} = 2.711731 \times 10^2$,

which is applied to the rate equation of Eq. B7. The apparent reaction Gibbs energy (Eq. A9) is obtained as

$\Delta_r G^{tot}_{fuma}(T, I) = -13.8882$ kJ/mol.

The biochemical reaction for the overall concentration of all species of reactant is

$FUM + H_2O = MAL$.

The reactant concentrations are denoted as [FUM] = substrateA and [MAL] = productP in the following enzymatic rate equation [1]:

J_{fuma} =

Vf*(substrateA-productP/Keq)/(KmA*((1+[CIT]/KiCIT+([ATP]/P_ATP4p*fP_ATP4p)/KiATP+([ADP]/P_A

DP3p*fP_ADP3p)/KiADP+([GTP]/P_GTP4p*fP_GTP4p)/KiGTP+([GDP]/P_GDP3p*fP_GDP3p)/KiGDP)+productP/KmP)+substrateA), (B7)

where P_ATP4p, P_ADP3p, P_GTP4p, and P_GDP3p are the binding polynomials for ATP^{4-}, ADP^{3}-, GTP^{4-}, and GDP^{3-}, respectively, provided by Eq. (A2), and fP_ATP4p, fP_ADP3p, fP_GTP4p, and fP_GDP3p are the corresponding binding polynomials from which all Mg^{2+}-binding species are excluded [1]. The parameters in Eq B7 are taken from the literature [1].

KmA	4.4700E-05	M
KmP	1.9770E-04	M
KiCIT	3.5000E-03	M
KiATP	4.0000E-05	M
KiADP	4.0000E-04	M
KiGTP	8.0000E-05	M
KiGDP	3.3000E-04	M

8. Malate dehydrogenase (EC: 1.1.1.37)

The reference reaction is

$NAD^{-} + MAL^{2-} = OAA^{2-} + NADH^{2-} + H^{+}$.

The standard-state reaction Gibbs free energy for the reference reaction (T_s = 298.15 K, I_s = 0 M) is calculated using the database, [2]

$\Delta_r G^0_{mdh}(T_s, I_s) = \Delta_f G^0_{\mathrm{OAA^{2-}}} + \Delta_f G^0_{\mathrm{NADH^{2-}}} + \Delta_f G^0_{\mathrm{H^+}} - \Delta_f G^0_{\mathrm{NAD^-}} - \Delta_f G^0_{\mathrm{MAL^{2-}}} = 71.08\ \mathrm{kJ/mol}.$

Using the reaction Gibbs free energy for the reference reaction (Eq. A3) at T = 298.15 K, pH = 7.5, I = 0.18 M, the apparent equilibrium constant (Eq. A7) is calculated as

$K_{eq}^{tot} = 7.105764 \times 10^{-5}$,

which is applied to the rate equation of Eq. B8. The apparent reaction Gibbs energy (Eq. A9) is obtained as

$\Delta_r G^{tot}_{mdh}(T, I) = 23.6777\ \mathrm{kJ/mol}.$

The biochemical reaction for the overall concentration of all species of reactant is

$NAD + MAL = OAA + NADH + H^{+}$.

The reactant concentrations are denoted as [NAD] = substrateA, [MAL] = substrateB, [OAA] = productP, and [NADH] = productQ in the following enzymatic rate equation [1]:

J_{mdh}= Vf*(substrateA*substrateB-productP*productQ/Keq)/(Kia*KmB*(1+(ATP/P_ATP4p*fP_ATP4p)/KiATP+(ADP/P_ADP3p*fP_ADP3p)/KiADP)+KmB*substrateA+KmA*substrateB*(1+(ATP/P_ATP4p*fP_ATP4p)/KiATP+(ADP/P_ADP3p*fP_ADP3p)/KiADP)+Vf_pVr_pKeq*KmQ*productP*(1+(ATP/P_ATP4p*fP_ATP4p)/KiATP+(ADP/P_ADP3p*fP_ADP3p)/KiADP)+Vf_pVr_pKeq*KmP*productQ+substrateA*substrateB+Vf_pVr_pKeq*KmQ*substrateA*productP/kia+Vf_pVr_pKeq*productP*productQ+KmA*substrate

B*productQ/Kiq+substrateA*substrateB*productP/Kip+Vf_pVr_pKeq*substrateB*productP*productQ/Kib),

(B8)

where P_ATP4p and P_ADP3p are the binding polynomials for ATP^{4-} and ADP^{3}-, respectively, that are provided by Eq. (A2), and fP_ATP4p and fP_ADP3p are the corresponding binding polynomials from which all Mg^{2+}-binding species are excluded [1]. A Haldane relation provides

Vf_pVr_pKeq = Vf/Vr/Keq = Kia*KmB/(KmP*Kiq) [10]. The parameters in Eq B8 are taken from the literature [1].

KmA	9.9550E-05	M
KmB	2.5000E-04	M
KmP	6.1280E-06	M
KmQ	2.5800E-06	M
Kia	2.7900E-04	M
Kib	3.6000E-04	M
Kip	5.5000E-06	M
Kiq	3.1800E-06	M
KiATP	1.8320E-04	M
KiADP	3.9440E-04	M
KiAMP	4.2000E-04	M

9. Pyruvate synthase (EC: 1.2.7.1) (or Pyruvate: ferredoxin oxidoreductase)

The reference reaction is

$2\ \mathrm{Fdx\_red}^{+} + \mathrm{ACOA}^{-} + \mathrm{CO_3}^{2-} + 2\ \mathrm{H}^{+} = 2\ \mathrm{Fdx\_ox}^{2+} + \mathrm{COA}^{-} + \mathrm{PYR}^{-} + \mathrm{H_2O}^{0}$.

The standard-state reaction Gibbs free energy for the reference reaction (T_s = 298.15 K, I_s = 0 M) is calculated using the database, [2]

$\Delta_r G^0_{pys}(T_s, I_s) = 2\Delta_f G^0_{\mathrm{Fdx\_ox^{2+}}} + \Delta_f G^0_{\mathrm{COA^-}} + \Delta_f G^0_{\mathrm{AKG^{2-}}} + \Delta_f G^0_{\mathrm{H_2O^0}} - 2\Delta_f G^0_{\mathrm{Fdx\_red^+}} - \Delta_f G^0_{\mathrm{SCOA^-}} - \Delta_f G^0_{\mathrm{CO_3^{2-}}} - 2\Delta_f G^0_{\mathrm{H^+}} = -66.98\ \mathrm{kJ/mol}$. As mentioned above, the standard-state Gibbs free energy change of ferredoxin (Fdx) upon the reduction is experimentally determined as $\Delta_r G^0_{\mathrm{Fdx\_red}} \equiv \Delta_f G^0_{\mathrm{Fdx\_red^+}} - \Delta_f G^0_{\mathrm{Fdx\_ox^{2+}}} = 36.7\ \mathrm{kJ/mol}$ [8].

Using the reaction Gibbs free energy for the reference reaction (Eq. A3) at T = 298.15 K, pH = 7.5, I = 0.18 M, the apparent equilibrium constant (Eq. A7) is calculated as

$K_{eq}^{tot} = 1.266899 \times 10^{-6}$,

which is applied to the rate equation of Eq. B9. The apparent reaction Gibbs energy (Eq. A9) is obtained as

$\Delta_r G^{tot}_{pys}(T, I) = 33.6597\ \mathrm{kJ/mol}$.

The biochemical reaction for the overall concentration of all species of reactant is

NAD + MAL = OAA + NADH + H^+.

The reactant concentrations are denoted as [Fdx_red] = substrateA, [ACOA] = substrateB, [CO_2(tot)] = substrateC, [Fdx_ox] = productP, [COA] = productQ, and [PYR] = productR in the following enzymatic rate equation [1]:

J_{pys} = Vf*(substrateA*substrateA*substrateB*substrateC-productP*productP*productQ*productR/Keq). (B9)

9'. Pyruvate dehydrogenase (EC: 1.2.1.aj)

The reference reaction is

$PYR^{-} + COA^{-} + NAD^{-} + H_2O^0 = CO_3^{2-} + ACOA^0 + NADH^{2-} + H^+$.

The standard-state reaction Gibbs free energy for the reference reaction (T_s = 298.15 K, I_s = 0 M) is calculated using the database, [2]

$\Delta_r G^0_{pdh}(T_s, I_s) = \Delta_f G^0_{\mathrm{CO_3^{2-}}} + \Delta_f G^0_{\mathrm{ACOA^0}} + \Delta_f G^0_{\mathrm{NADH^{2-}}} + \Delta_f G^0_{\mathrm{H^+}} - \Delta_f G^0_{\mathrm{PYR^-}} - \Delta_f G^0_{\mathrm{COA^-}} - \Delta_f G^0_{\mathrm{NAD^-}} - \Delta_f G^0_{\mathrm{H_2O^0}} =$ 17.49 kJ/mol.

Using the reaction Gibbs free energy for the reference reaction (Eq. A3) at T = 298.15 K, pH = 7.5, I = 0.18 M, the apparent equilibrium constant (Eq. A7) is calculated as

$K^{tot}_{eq} = 1.82773 \times 10^9$,

which is applied to the rate equation of Eq. B9'. The apparent reaction Gibbs energy (Eq. A9) is obtained as

$\Delta_r G^{tot}_{pdh}(T, I) = -52.86$ kJ/mol.

The biochemical reaction for the overall concentration of all species of reactant is

PYR + COA + H_2O + NAD = ACOA + CO_2(tot) + NADH + H^+.

The reactant concentrations are denoted as [PYR] = substrateA, [COA] = substrateB, [NAD] = substrateC, [CO_2(tot)] = productP, [ACOA] = productQ, and [NADH] = productR in the following enzymatic rate equation [1]:

J_{pdh} =

Vf*(substrateA*substrateB*substrateC-productP*productQ*productR/Keq)/(KmC*substrateA*substrateB*(1+productR/KiNADH)+KmB*substrateA*substrateC*(1+productQ/KiACOA)+KmA*substrateB*substrateC+substrateA*substrateB*substrateC). (B9')

The parameters in Eq B9' are taken from the literature [1].

KmA	3.8300E-05	M
KmB	9.9000E-06	M
KmC	6.0700E-05	M
KiACOA	4.0200E-05	M
KiNADH	4.0000E-05	M

The reactions associated with TCA cycle including anaplerotic reactions

10. NADP-Malic enzyme (EC: 1.1.1.40)

The reference reaction is

$NADP^{3-} + MAL^{2-} + H_2O^0 = CO_3^{2-} + PYR^{-} + NADPH^{4-} + 2 H^+$.

The standard-state reaction Gibbs free energy for the reference reaction (T_s = 298.15 K, I_s = 0 M) is calculated using the database, [2]

$$\Delta_r G^0_{me}(T_s, I_s) = \Delta_f G^0_{CO_3^{2-}} + \Delta_f G^0_{PYR^{2-}} + \Delta_f G^0_{NADPH^{4-}} + 2\Delta_f G^0_{H^+} - \Delta_f G^0_{NADP^{3-}} - \Delta_f G^0_{MAL^{2-}} - \Delta_f G^0_{H_2O^0} = 103.45 \text{ kJ/mol}.$$

Using the reaction Gibbs free energy for the reference reaction (Eq. A3) at T = 298.15 K, pH = 7.5, I = 0.18 M, the apparent equilibrium constant (Eq. A7) is calculated as

$$K_{eq}^{tot} = 7.918251 \times 10^1,$$

which is applied to the rate equation of Eq. B10. The treatment of CO_2 in Eq. A7 will be mentioned below. The apparent reaction Gibbs energy (Eq. A9) is obtained as

$$\Delta_r G^{tot}_{me}(T, I) = -10.83679 \text{ kJ/mol}.$$

The biochemical reaction for the overall concentration of all species of reactant is

NADP + MAL + H_2O = CO_2(tot) + PYR + NADPH + 2 H^+.

The kinetic model for malic enzyme was developed using the ordered bi-ter mechanism [10]. The parameters in the ordered bi-ter rate equation were in part determined so that it reproduced available experimental data for a NAD-dependent malic enzyme [11].

The reactant concentrations are denoted as [NADP]=substrateA, [MAL]=substrateB, [CO_2(tot)]=productP, [PYR]=productQ, and [NADPH]=productR in the following enzymatic rate equation:

J_{me} = Vf*(substrateA*substrateB -productP*f_H2CO3*productQ*productR/Keq)/(Kia*Kb +Kb*substrateA +Ka*substrateB +substrateA*substrateB+alpha_Kq_Kir*productP*f_H2CO3 +alpha_Kp_Kiq*productR +alpha_Kq_Kir/Kia*substrateA*productP*f_H2CO3 +Ka/Kir*substrateB*productR +alpha_Kq_Kir/(Kia*Kib)*substrateA*substrateB*productP*f_H2CO3 +alpha_Kr_Kip/(Kia*Kib)*substrateA*substrateB*productQ), (B10)

where f_H2CO3 is the fraction of H_2CO_3, thereby in Eq. A7, the binding polynomial for CO_3^{2-} is replaced with that multiplied by f_H2CO3.

The parameters in Eq B10 are determined using the biochemical experimental data [11].

Ka	2.2592E-04	M
Kb	7.1082E-03	M
Kia	1.1724E-03	M
Kib	4.7942E-02	M
Kp		M
Kq		M
Kr		M
Kip		M

Kiq		M
Kir	1.7202E-02	M
Alpha=Vf/(Vr*Keq)		1/M
alpha_Kq_Kir = alpha*Kq*Kir	3.9889E-02	M
alpha_Kr_Kip = alpha*Kr*Kip	8.0236E-03	M
alpha_Kp_Kiq = alpha*Kp*Kiq	4.9126E-02	M
f_H2CO3	0.042596084	1

11. Phosphoenolpyruvate synthase (EC: 2.7.9.2)

The reference reaction is

$ATP^{4-} + PYR^{-} + H_2O^0 = AMP^{2-} + Pi^{2-} + PEP^{3-} + 2\ H^+$.

The standard-state reaction Gibbs free energy for the reference reaction (T_s = 298.15 K, I_s = 0 M) is calculated using the database, [2]

$\Delta_r G^0_{pps}(T_s, I_s) = \Delta_f G^0_{\mathrm{AMP^{2-}}} + \Delta_f G^0_{\mathrm{Pi^{2-}}} + \Delta_f G^0_{\mathrm{PEP^{3-}}} + 2\Delta_f G^0_{\mathrm{H^+}} - \Delta_f G^0_{\mathrm{ATP^{4-}}} - \Delta_f G^0_{\mathrm{PYR^-}} - \Delta_f G^0_{\mathrm{H_2O^0}} = 79.46$ kJ/mol.

Using the reaction Gibbs free energy for the reference reaction (Eq. A3) at T = 298.15 K, pH = 7.5, I = 0.18 M, the apparent equilibrium constant (Eq. A7) is calculated as

$K_{eq}^{tot} = 6.876024 \times 10^2$,

which is applied to the rate equation of Eq. B11. The apparent reaction Gibbs energy (Eq. A9) is obtained as

$\Delta_r G_{pps}^{tot}(T, I) = -18.63$ kJ/mol.

The biochemical reaction for the overall concentration of all species of reactant is

$ATP + PYR + H_2O = AMP + Pi + PEP + 2\ H^+$.

The kinetic model for Phosphoenolpyruvate synthase was developed. The parameters in the rate equation were in part determined so that it reproduced available experimental data for Phosphoenolpyruvate synthase of *E. coli* [12].

The reactant concentrations are denoted as [ATP]=substrateA, [PYR]=substrateB, [AMP]=productP, [Pi]=productQ, and [PEP]=productR in the following enzymatic rate equation:

J_{pps} = Vf*(substrateA*substrateB-productP*productQ*productR/Keq)/(KMg/[Mg^{2+}]*Kib*substrateA +(1+KiMg/[Mg^{2+}])*Ka*substrateB +(1+KMg/[Mg^{2+}])*substrateA*substrateB). (B11)

The parameters in Eq B11 are determined using the biochemical experimental data [12].

Ka	1.2964E-05	M
Kb	0.0000E+00	M

Kib	4.4367E-04	M
KMg	3.8357E-03	M
KiMg	3.5791E-02	M

12. Phosphoenolpyruvate Carboxykinase (EC: 4.1.1.49)

The reference reaction is

$ATP^{4-} + OAA^{2-} + H_2O^0 = CO_3^{2-} + PEP^{3-} + ADP^{3-} + 2\ H^+$.

The standard-state reaction Gibbs free energy for the reference reaction (T_s = 298.15 K, I_s = 0 M) is calculated using the database, [2]

$$\Delta_r G^0_{ppck}(T_s, I_s) = \Delta_f G^0_{\mathrm{CO_3^{2-}}} + \Delta_f G^0_{\mathrm{PEP^{3-}}} + \Delta_f G^0_{\mathrm{ADP^{3-}}} + 2\Delta_f G^0_{\mathrm{H^+}} - \Delta_f G^0_{\mathrm{ATP^{4-}}} - \Delta_f G^0_{\mathrm{OAA^{2-}}} - \Delta_f G^0_{\mathrm{H_2O^0}} = 95.69\ \mathrm{kJ/mol}.$$

Using the reaction Gibbs free energy for the reference reaction (Eq. A3) at T = 298.15 K, pH = 7.5, I = 0.18 M, the apparent equilibrium constant (Eq. A7) is calculated as

$K_{eq}^{tot} = 9.534605 \times 10^2$,

which is applied to the rate equation of Eq. B12. The apparent reaction Gibbs energy (Eq. A9) is obtained as

$\Delta_r G_{ppck}^{tot}(T, I) = -17.00\ \mathrm{kJ/mol}$.

The biochemical reaction for the overall concentration of all species of reactant is

$ATP + OAA + H_2O = CO_2(tot) + PEP + ADP + 2\ H^+$.

The kinetic model for Phosphoenolpyruvate Carboxykinase was developed using the ordered bi-ter mechanism [10]. The parameters in the enzymatic rate equation were in part determined so that it reproduced available experimental data for Phosphoenolpyruvate Carboxykinase of *E. coli* [13].

The reactant concentrations are denoted as [ATP]=substrateA, [OAA]=substrateB, [CO_2(tot)]=productP, [PEP]=productQ, and [ADP]=productR in the following enzymatic rate equation:

J_{ppck} = Vf*(substrateA*substrateB -productP*productQ*productR/Keq)/(Kia*Kb +Kb*substrateA +Ka*substrateB +substrateA*substrateB+alpha_Kq*Kir*productP +alpha_Kp*Kiq*productR +alpha_Kr*productP*productQ +alpha_Kq*productP*productR +alpha_Kp*productQ*productR +alpha*productP*productQ*productR +alpha_Kq*Kir/Kia*substrateA*productP +alpha_Kr/Kia*substrateA*productP*productQ). (B12)

The parameters in Eq B12 are determined using the biochemical experimental data [12].

Ka	4.0000E-01	M
Kb	1.3186E-03	M
Kia	7.6387E-04	M
Kib	2.0000E-03	M
alpha_Kp = alpha*Kp	1.3162E-01	1
alpha_Kq = alpha*Kq	5.2467E-03	1

alpha_Kr = alpha*Kr	5.6652E-07	1
Kip	non	M
Kiq	3.4401E-03	M
Kir	1.8907E-02	M
alpha=Vf/(Vr*Keq)	4.4702E+00	1/M

13. Pyruvate Kinase (EC: 2.7.1.40)

The reference reaction is

$PYR^{-} + ATP^{4-} = PEP^{3-} + ADP^{3-} + H^{+}$.

The standard-state reaction Gibbs free energy for the reference reaction (T_s = 298.15 K, I_s = 0 M) is calculated using the database, [2]

$\Delta_r G^0_{pyk}(T_s, I_s) = \Delta_f G^0_{\mathrm{PEP^{3-}}} + \Delta_f G^0_{\mathrm{ADP^{3-}}} + \Delta_f G^0_{\mathrm{H^+}} - \Delta_f G^0_{\mathrm{ATP^{4-}}} - \Delta_f G^0_{\mathrm{PYR^-}} = 66.9$ kJ/mol.

Using the reaction Gibbs free energy for the reference reaction (Eq. A3) at T = 298.15 K, pH = 7.5, I = 0.18 M, the apparent equilibrium constant (Eq. A7) is calculated as

$K^{tot}_{eq} = 9.265153 \times 10^{-5}$,

which is applied to the rate equation of Eq. B13. The apparent reaction Gibbs energy (Eq. A9) is obtained as

$\Delta_r G^{tot}_{pyk}(T, I) = 23.02$ kJ/mol.

The biochemical reaction for the overall concentration of all species of reactant is

ATP + PYR = PEP + ADP + H^{+}.

The kinetic model for Pyruvate kinase was developed using the rapid-equilibrium random bi-bi mechanism [10]. The parameters in the enzymatic rate equation were in part determined so that it reproduced available experimental data for Pyruvatr kinase of rabbit muscle [14].

The reactant concentrations are denoted as [PYR] = substrateA, [ATP] =s ubstrateB, [PEP] = productP, and [ADP] = productQ in the following enzymatic rate equation:

J_{pyk} = Vf*(substrateA*substrateB-productP*productQ/Keq)/(Kia*Kb +Kb*substrateA + Ka*substrateB + substrateA*substrateB+Kia*Kb/Kiq*Kq_pKp*productP + Kia*Kb/Kiq*productQ). (B13)

The parameters in Eq B13 are determined using the biochemical experimental data [14].

Ka	6.8377E-03	M
Kb	1.8627E-04	M
Kia	8.3501E-03	M
Kiq	2.1275E-05	M
Kq_pKp = Kq/Kp	1.8891E+00	1
Kp	non	M

14. Pyruvate Carboxylase (EC: 6.4.1.1)

The reference reaction is

$ATP^{4-} + CO_3^{2-} + PYR^{-} = Pi^{2-} + ADP^{3-} + OAA^{2-}$.

The standard-state reaction Gibbs free energy for the reference reaction (T_s = 298.15 K, I_s = 0 M) is calculated using the database, [2]

$$\Delta_r G^0_{pyc}(T_s, I_s) = \Delta_f G^0_{\mathrm{Pi^{2-}}} + \Delta_f G^0_{\mathrm{ADP^{3-}}} + \Delta_f G^0_{\mathrm{OAA^{2-}}} - \Delta_f G^0_{\mathrm{ATP^{4-}}} - \Delta_f G^0_{\mathrm{CO_3^{2-}}} - \Delta_f G^0_{\mathrm{PYR^{-}}} = -24.12\ \mathrm{kJ/mol}.$$

Using the reaction Gibbs free energy for the reference reaction (Eq. A3) at T = 298.15 K, pH = 7.5, I = 0.18 M, the apparent equilibrium constant (Eq. A7) is calculated as

$$K^{tot}_{eq} = 1.087363,$$

which is applied to the rate equation of Eq. B14. The apparent reaction Gibbs energy (Eq. A9) is obtained as

$$\Delta_r G^{tot}_{pyc}(T, I) = -0.21\ \mathrm{kJ/mol}.$$

The biochemical reaction for the overall concentration of all species of reactant is

$ATP + CO_2(tot) + PYR = Pi + ADP + OAA$.

The kinetic model for Pyruvate carboxylase of chicken liver was developed using a modified Ping-Pong Bi Bi mechanism [15], where the parameters in the enzymatic rate equation were determined so that it reproduced available experimental data [15].

The reactant concentrations are denoted as [ATP] = substrateA, [CO_2(tot)] =substrateB, [PYR] =substrateC, [Pi] = productP, [ADP] = productQ, and [OAA] = productR in the following enzymatic rate equation:

J_{pyc} = Vf*(substrateA *substrateB *substrateC-productP*productQ*productR/Keq)/(Kia*Kb*substrateC +Kc*substrateA*substrateB+Ka*substrateB*substrateC +Kb*substrateA*substrateC +substrateA*substrateB*substrateC +Kip*Kq*productR +Kq*productP*productR +Kp*productQ*productR +Kr*productP*productQ +alpha*productP*productQ*productR +Kia*Kb/Kip*substrateC*productP +Kia*Kb/Kiq*substrateC*productQ +Kiq*Kp/Kib*substrateB*productR +Kiq*Kp/Kia*substrateA*productR +Kc/Kir*substrateA*substrateB*productR +Ka/Kiq*substrateB*substrateC*productQ +Ka/Kip*substrateB*substrateC*productP +Kp/Kib*substrateB*productQ*productR +Kq/Kib*substrateB*productP*productR). (B14)

The parameters in Eq. B14 are determined using the biochemical experimental data [15].

Ka	2.2868E-04	M
Kb	2.0972E-03	M
Kc	1.4279E-03	M
Kia	1.4832E-04	M
Kib	1.1373E-02	M
Kic	no data	M
Kp	7.4351E-03	M
Kq	2.8252E-03	M
Kr	7.9964E-05	M

Kip	1.2389E-02	M
Kiq	2.9444E-09	M
Kir	2.1234E-03	M
Alpha = Vf/(Vr*Keq)	assumed to be 1	1/M

The other reactions

15. Ferredoxin—NAD reductase (EC: 1.18.1.3)

The reference reaction is

$NADH^{2-} + 2\ Fdx\_ox^{2+} = NAD^{-} + 2\ Fdx\_red^{+} + H^{+}$.

The standard-state reaction Gibbs free energy for the reference reaction (T_s = 298.15 K, I_s = 0 M) is calculated using the database, [2]

$\Delta_r G^0_{fdx\_nad}(T_s, I_s) = \Delta_f G^0_{\mathrm{NAD^-}} + 2\Delta_f G^0_{\mathrm{Fdx\_red^+}} + \Delta_f G^0_{\mathrm{H^+}} - \Delta_f G^0_{\mathrm{NADH^{2-}}} + 2\Delta_f G^0_{\mathrm{Fdx\_ox^{2+}}} = 49.49\ \mathrm{kJ/mol}$.

As mentioned above, the standard-state Gibbs free energy change of Fdx upon the reduction is experimentally determined as $\Delta_r G^0_{\mathrm{Fdx\_red}} \equiv \Delta_f G^0_{\mathrm{Fdx\_red^+}} - \Delta_f G^0_{\mathrm{Fdx\_ox^{2+}}} = 36.7\ \mathrm{kJ/mol}$ [8].

Using the reaction Gibbs free energy for the reference reaction (Eq. A3) at T = 298.15 K, pH = 7.5, I = 0.18 M, the apparent equilibrium constant (Eq. A7) is calculated as

$K_{eq}^{tot} = 4.318638 \times 10^{-4}$,

which is applied to the rate equation of Eq. B15. The apparent reaction Gibbs energy (Eq. A9) is obtained as

$\Delta_r G^{tot}_{fdx\_nad}(T, I) = 19.20\ \mathrm{kJ/mol}$.

The biochemical reaction for the overall concentration of all species of reactant is

NADH + 2 Fdx_ox = NAD + 2 Fdx_red + H^{+}.

The enzymatic rate equation is as follows:

$J_{\mathrm{fdx\_nad}}$ = Vf*(NADH*Fdx_ox*Fdx_ox-NAD*Fdx_red*Fdx_red/Keq). (B15)

16. Ferredoxin−NADP reductase (EC: 1.18.1.7)

The reference reaction is

$NADPH^{4-} + 2\ Fdx\_ox^{2+} = NAPD^{3-} + 2\ Fdx\_red^{+} + H^{+}$.

The standard-state reaction Gibbs free energy for the reference reaction (T_s = 298.15 K, I_s = 0 M) is calculated using the database, [2]

$\Delta_r G^0_{fdx\_nadp}(T_s, I_s) = \Delta_f G^0_{\mathrm{NADP^{3-}}} + 2\Delta_f G^0_{\mathrm{Fdx\_red^+}} + \Delta_f G^0_{\mathrm{H^+}} - \Delta_f G^0_{\mathrm{NADPH^{4-}}} + 2\Delta_f G^0_{\mathrm{Fdx\_ox^{2+}}} = 45.91\ \mathrm{kJ/mol}$.

As mentioned above, the standard-state Gibbs free energy change of Fdx upon the reduction is experimentally determined as $\Delta_r G^0_{\mathrm{Fdx\_red}} \equiv \Delta_f G^0_{\mathrm{Fdx\_red^+}} - \Delta_f G^0_{\mathrm{Fdx\_ox^{2+}}} = 36.7\ \mathrm{kJ/mol}$ [8].

Using the reaction Gibbs free energy for the reference reaction (Eq. A3) at T = 298.15 K, pH = 7.5, I = 0.18 M, the apparent equilibrium constant (Eq. A7) is calculated as

$K_{eq}^{tot} = 1.698823 \times 10^{-4}$,

which is applied to the rate equation of Eq. B16. The apparent reaction Gibbs energy (Eq. A9) is obtained as $\Delta_r G^{tot}_{fdx\_nadp}(T, I) = 21.52$ kJ/mol.

The biochemical reaction for the overall concentration of all species of reactant is

NADPH + 2 Fdx_ox = NADP + 2 Fdx_red + H^+.

The enzymatic rate equation is as follows:

$J_{\mathrm{fdx\_nadp}}$ = Vf*(NADPH*Fdx_ox*Fdx_ox-NADP*Fdx_red*Fdx_red/Keq). (B16)

17. Adenylate kinase (EC: 2.7.4.3)

The reference reaction is

$AMP^{2-} + ATP^{4-} = 2\ ADP^{3-}$.

The standard-state reaction Gibbs free energy for the reference reaction (T_s = 298.15 K, I_s = 0 M) is calculated using the database, [2]

$\Delta_r G^0_{adk}(T_s, I_s) = 2\Delta_f G^0_{\mathrm{ADP^{3-}}} - \Delta_f G^0_{\mathrm{AMP^{2-}}} - \Delta_f G^0_{\mathrm{ATP^{4-}}} = -7.89$ kJ/mol.

Using the reaction Gibbs free energy for the reference reaction (Eq. A3) at T = 298.15 K, pH = 7.5, I = 0.18 M, the apparent equilibrium constant (Eq. A7) is calculated as

$K^{tot}_{eq} = 2.483758$,

which is applied to the rate equation of Eq. B17. The apparent reaction Gibbs energy (Eq. A9) is obtained as $\Delta_r G^{tot}_{adk}(T, I) = -2.26$ kJ/mol.

The biochemical reaction for the overall concentration of all species of reactant is

AMP + ATP = 2 ADP.

The enzymatic rate equation is as follows:

J_{adk} = Vf*(AMP*ATP-ADP*ADP/Keq). (B17)

18. Biomass synthesis

The biochemical reaction for the overall concentration of all species of reactant is [16]

N_{OAA} OAA + N_{AKG} AKG + N_{PYR} PYR + N_{PEP} PEP + N_{NADPH} NADPH + N_{ACOA} ACOA + N_{NAD} NAD + N_{ATP} ATP ->,

where N_{OAA} = 1010, N_{AKG} = 610, N_{PYR} = 1601, N_{PEP} = 293, N_{NADPH} = 10169, N_{ACOA} = 2118, N_{NAD} = 2004, N_{ATP} = 30508. The rate equation for species i is as follows:

$J_{\mathrm{growth}}(i)$ =

N_i*V_{growth}*OAA*AKG*PYR*PEP*NADPH*ACOA*NAD*ATP/(KmOAA*KmAKG*KmPYR*KmPEP*KmNADPH*KmACOA*KmNAD*KmATP)/((1+OAA/KmOAA)*(1+AKG/KmAKG)*(1+PYR/KmPYR)*(1+PEP/KmPEP)*(1+NADPH/KmNADPH)*(1+ACOA/KmACOA)*(1+NAD/KmNAD)*(1+ATP/KmATP)).

(B18)

The parameters in Eq. B18 are taken from the literature [16].

KmOAA	2.48e-5	M
KmAKG	0.00521	M
KmPYR	4.64e-6	M
KmPEP	0.000458	M
KmNADPH	0.003598	M
KmACOA	4.94e-5	M
KmNAD	0.002822	M
KmATP	4.68e-5	M

Table A1. The model parameters used in the kinetic network model simulation for all the organisms of which results are provided in the main text and the SI. In the cases with a complete oTCA cycle (Figs. S5d and S6f), Fdx_red and Fdx_ox, which control the concentration ratio of reducing agents to oxidative agents, i.e., [NADH]/[NAD] and [NADPH]/[NADP], were varied so that the kinetic network model simulated the oTCA cycle.

Parameter	Value	Units	References and explanations
NAD_{tot}	0.00157	M	[17,18]
$NADP_{tot}$	0.0011	M	[17,18]
Fdx_ox	5e-8	M	Estimated so that the kinetic network model reproduces the concentrations for autotrophically grown *D. acetivorans* (Table S1) [19].
Fdx_red	0.0025	M	Estimated so that the kinetic network model reproduces the concentrations for autotrophically grown *D. acetivorans* (Table S1) [19].
ATP	0.003	M	This work. ATP_{tot} taken from [17,18].
ADP	0.00148	M	This work. ATP_{tot} taken from [17,18].
Pi	0.005	M	[18,20]
GTP	0.003	M	Assumed in this work.
GDP	0.00148	M	Assumed in this work.
COQ	2e-7	M	Estimated so that the kinetic network model reproduces the concentrations for autotrophically grown *D. acetivorans* (Table S1) [19].
QH_2	0.0007	M	Estimated so that the kinetic network model reproduces the concentrations for autotrophically grown *D. acetivorans* (Table S1) [19]. This is close to 1 mM [17,18].
COA	0.001	M	Experimentally determined for autotrophically grown *D.*

			acetivorans [19].
CO_2(tot)	0.0214	M	[1,21]
Mg^{2+}	0.0008	M	[2]
pH	7.5		[18,20]

Table A2. Maximum flux parameters for enzymatic reactions V_f divided by maximum flux parameter for biosynthesis V_{growth} (Eq. B18) are provided below.

**Enzyme**	$V_f/V_{growth} \times 10^{11}$
1. Citrate synthase	5.0000
2. Aconitate	0.0500
3. Isocitrate dehydrogenase	5.0000
4. 2-oxoglutarate synthase	5.0000
5. Succinyl-CoA synthetase	0.0500
6. Succinate dehydrogenase	0.0500
7. Fumarate hydratase	0.0500
8. Malate dehydrogenase	5.0000
9. Pyruvate synthase	5.0000
10. NADP-Malic enzyme	0.0005
11. Phosphoenolpyruvate synthase	0.0005
12. Phosphoenolpyruvate Carboxykinase	0.0005
13. Pyruvate Kinase	0.0005
14. Pyruvate Carboxylase	0.0005
15. Ferredoxin—NAD reductase	0.0500
16. Ferredoxin−NADP reductase	0.0500
17. Adenylate kinase	0.0500

Table S1a. The steady-state fluxes for *T. takaii* grown chemolithoautotrophically obtained from the kinetic network model. The fixed model parameters used here are listed in Table A1. In the column of reaction, the chemical species following the semicolon indicate the effector compounds involved in the reaction rate equation. The positive value of the flux corresponds to the positive direction of the reaction equation listed in this table and of the flux shown in Fig A1.

	NAME	FLUX [MMOL/S]	REACTION
1	Mdh	-3.66E-06	NAD + MAL = OAA + NADH; ATP ADP
2	Fuma	-2.34E-06	FUM = MAL; CIT ATP ADP GTP GDP
3	Sdh	-2.34E-06	SUC + COQ = QH2 + FUM; OAA SUC FUM
4	Scas_ADPform	-2.34E-06	ADP + SCOA + Pi = COAS + SUC + ATP
5	AKG_Fdx	2.34E-06	2 * Fdx_red + SCOA + CO2 = 2 * Fdx_ox + COAS + AKG
6	Icdh_NADPform	-2.12E-06	NADP + ICIT = NADPH + AKG + CO2; H+
7	Ac	-2.12E-06	CIT = ICIT
8	Cs	-2.12E-06	OAA + ACOA = COAS + CIT; H+ ATP ADP SCOA
9	Pys_Fdx	1.32E-06	2 * Fdx_red + ACOA + CO2 = 2 * Fdx_ox + COAS + PYR
10	Me_NADPform	1.32E-06	NADP + MAL = CO2 + PYR + NADPH
11	Pck	1.26E-08	ATP + OAA = CO2 + PEP + ADP
12	Pyk	-9.78E-07	PYR + ATP = PEP + ADP
13	Pyc	1.94E-06	ATP + CO2 + PYR = Pi + ADP + OAA
14	Pps	1.08E-06	ATP + PYR = AMP + Pi + PEP; Mg
15	GROWTH by OAA	3.79E-07	OAA → ; AKG PYR PEP NADPH ACOA NAD ATP
16	GROWTH by AKG	2.29E-07	AKG → ; OAA PYR PEP NADPH ACOA NAD ATP
17	GROWTH by PYR	6.01E-07	PYR → ; OAA AKG PEP NADPH ACOA NAD ATP
18	GROWTH by PEP	1.10E-07	PEP →; OAA AKG PYR NADPH ACOA NAD ATP
19	GROWTH by ACOA	7.95E-07	ACOA → ; OAA AKG PYR PEP NADPH NAD ATP
20	Adk	1.08E-06	AMP + ATP = 2 * ADP
21	FdxR_NAD	-3.66E-06	NADH + 2 * Fdx_ox = NAD + 2 * Fdx_red
22	FdxR_NADP	-7.99E-07	NADPH + 2 * Fdx_ox = NADP + 2 * Fdx_red

Table S1b. The steady-state fluxes for *T. takaii* with the gene knockouts of malic enzyme between MAL and PYR and of Phosphoenolpyruvate Carboxykinase between OAA and PEP obtained from the kinetic network model. The fixed model parameters used here are listed in Table A1. In the column of reaction, the chemical species following the semicolon indicate the effector compounds involved in the reaction rate equation. The positive value of the flux corresponds to the positive direction of the reaction equation listed in this table and of the flux shown in Fig A1.

	NAME	FLUX [MMOL/S]	REACTION
1	Mdh	-4.44E-06	NAD + MAL = OAA + NADH; ATP ADP
2	Fuma	-4.44E-06	FUM = MAL; CIT ATP ADP GTP GDP
3	Sdh	-4.44E-06	SUC + COQ = QH2 + FUM; OAA SUC FUM
4	Scas_ADPform	-4.44E-06	ADP + SCOA + Pi = COAS + SUC + ATP
5	AKG_Fdx	4.44E-06	2 * Fdx_red + SCOA + CO2 = 2 * Fdx_ox + COAS + AKG
6	Icdh_NADPform	-4.00E-06	NADP + ICIT = NADPH + AKG + CO2; H+
7	Ac	-4.00E-06	CIT = ICIT
8	Cs	-4.00E-06	OAA + ACOA = COAS + CIT; H+ ATP ADP SCOA
9	Pys_Fdx	2.50E-06	2 * Fdx_red + ACOA + CO2 = 2 * Fdx_ox + COAS + PYR
10	Me_NADPform	0.00E+00	NADP + MAL = CO2 + PYR + NADPH
11	Pck	0.00E+00	ATP + OAA = CO2 + PEP + ADP
12	Pyk	-4.33E-07	PYR + ATP = PEP + ADP
13	Pyc	1.15E-06	ATP + CO2 + PYR = Pi + ADP + OAA
14	Pps	6.41E-07	ATP + PYR = AMP + Pi + PEP; Mg
15	GROWTH by OAA	7.18E-07	OAA → ; AKG PYR PEP NADPH ACOA NAD ATP
16	GROWTH by AKG	4.33E-07	AKG → ; OAA PYR PEP NADPH ACOA NAD ATP
17	GROWTH by PYR	1.14E-06	PYR → ; OAA AKG PEP NADPH ACOA NAD ATP
18	GROWTH by PEP	2.08E-07	PEP → ; OAA AKG PYR NADPH ACOA NAD ATP
19	GROWTH by ACOA	1.50E-06	ACOA → ; OAA AKG PYR PEP NADPH NAD ATP
20	Adk	6.41E-07	AMP + ATP = 2 * ADP
21	FdxR_NAD	-4.44E-06	NADH + 2 * Fdx_ox = NAD + 2 * Fdx_red
22	FdxR_NADP	-4.00E-06	NADPH + 2 * Fdx_ox = NADP + 2 * Fdx_red

Table S2. The concentrations of metabolites in the rTCA cycle simulated by the kinetic network model with the fixed concentrations that are listed in Table A1. The metabolite concentrations experimentally determined are for *D. acetivorans* grown chemolithoautotrophically [19].

Metabolite	Simulation, mM	Experiment, mM
Acetyl-CoA	0.099	0.012
Citrate	1.55	1.43
Succinate	3.09	2.91
Malate	0.60	0.30

Table S3. The apparent reaction Gibbs energy $\Delta_r G_i^{tot}$ defined by Eq. A9 at the thermodynamic state, *T* = 298.15 K, pH = 7.5, *I* = 0.18 M. $\Delta_r G_i^{tot}$ was calculated using Eqs. A2–A9, where the standard-state Gibbs free energy of formation $\Delta_f G_i^0(T_s, I_s)$ for reference species [2,5] was used in Eq. A3. In the reactions 4, 9, 15, and 16, the standard-state Gibbs free energy change of ferredoxin (Fdx) upon the reduction $\Delta_r G^0_{\text{Fdx\_red}} \equiv \Delta_f G^0_{\text{Fdx\_red}} - \Delta_f G^0_{\text{Fdx\_ox}}$ was experimentally determined to be 36.7 kJ/mol [8].

Enzyme (EC number)	$\Delta_r G_i^{tot}$ (kJ/mol)	Reaction equation with the total concentration of each species
**TCA cycle**		
**Total**: Oxidative TCA with Fdx_red	−108.76	$\Delta_r G_1^{tot} + \Delta_r G_2^{tot} + \Delta_r G_3^{tot} + \Delta_r G_4^{tot} + \Delta_r G_5^{tot} + \Delta_r G_6^{tot} + \Delta_r G_7^{tot} + \Delta_r G_8^{tot} - \Delta_r G_{14}^{tot} + \Delta_r G_9^{tot}$
**Total**: Oxidative TCA with NADH	−147.16	$\Delta_r G_1^{tot} + \Delta_r G_2^{tot} + \Delta_r G_3^{tot} + \Delta_r G_4^{tot} + \Delta_r G_5^{tot} + \Delta_r G_6^{tot} + \Delta_r G_7^{tot} + \Delta_r G_8^{tot} - \Delta_r G_{14}^{tot} + \Delta_r G_{9'}^{tot}$
1. Citrate synthase (EC:2.3.3.1)	−53.43	OAA + ACOA + H_2O = COA + CIT + 2 H^+
2. Aconitate (EC: 4.2.1.3)	8.56	CIT = ICIT
3. Isocitrate dehydrogenase (EC:1.1.1.42)	−0.12	ICIT + NADP + H_2O = AKG + NADPH + CO_2(tot) + 2 H^+
4. 2-oxoglutarate synthase (EC: C1.2.7.3) (2-oxoglutarate: ferredoxin oxidoreductase)	−32.96	AKG + COA + H_2O + 2 Fdx_ox = SCOA + CO_2(tot) + 2 Fdx_red + 2 H^+
4’. 2-oxoglutarate dehydrogenase (EC: 1.2.1.ak)	−52.16	AKG + COA + H_2O + NAD = SCOA + CO_2(tot) + NADH + H^+
5. Succinyl-CoA synthetase (EC: 6.2.1.5)	−4.88	SCOA + ADP + Pi = SUC + COA + ATP + H^+

6. Succinate dehydrogenase (EC: 1.3.5.1)	−2.27	SUC + COQ = FUM + QH_2
7. Fumarate hydratase (EC: 4.2.1.2)	−13.89	FUM + H_2O = MAL
8. Malate dehydrogenase (EC: 1.1.1.37)	23.68	MAL + NAD = OAA + NADH + H^+
9. Pyruvate synthase (EC: 1.2.7.1) (Pyruvate: ferredoxin oxidoreductase)	−33.66	PYR + COA + H_2O + 2 Fdx_ox = ACOA + CO_2(tot) + 2 Fdx_red + 2 H^+
9'. Pyruvate dehydrogenase (EC: 1.2.1.aj)	−52.86	PYR + COA + H_2O + NAD = ACOA + CO_2(tot) + NADH + H^+
**Its associated reactions including anaplerotic ones**		
10. NADP-Malic enzyme (EC: 1.1.1.40)	−10.84	MAL + H_2O + NADP = PYR + CO_2(tot) + NADPH + 2 H^+
11. Phosphoenolpyruvate synthase (EC: 2.7.9.2)	−18.63	PYR + ATP + H_2O = PEP + AMP + Pi + 2 H^+
12. Phosphoenolpyruvate Carboxykinase (EC: 4.1.1.49)	−17.00	OAA + ATP + H_2O = PEP + CO_2(tot) + ADP + 2 H^+
13. Pyruvate Kinase (EC: 2.7.1.40)	23.02	PYR + ATP = PEP + ADP + H^+
14. Pyruvate Carboxylase (EC: 6.4.1.1)	−0.21	PYR + CO_2(tot) + ATP = OAA + ADP + Pi
**The other reactions**		
15. Ferredoxin−NAD reductase (EC: 1.18.1.3)	19.20	NADH + 2 Fdx_ox = NAD + 2 Fdx_red + H^+
16. Ferredoxin−NADP reductase (EC: 1.18.1.7)	21.52	NADPH + 2 Fdx_ox = NADP + 2 Fdx_red + H^+
17. Adenylate kinase (EC: 2.7.4.3)	−2.26	AMP + ATP = 2 ADP

Table S4. The role of pyruvate carboxylase and pyruvate synthase on the rTCA cycle. The directions of steady state fluxes for *T. takaii*-type *and D. acetivoransi*-type model are shown in Fig. S5e and S5f, respectively. *D. acetivoransi* possesses an enzyme of serine metabolism which provides a pyruvate influx into the rTCA cycle, while *T. takaii* does not. In the knockout simulations of pyruvate carboxylase, both the models totally impaired the rTCA flux. On the other hand, in the knockout simulations of pyruvate synthase, the *D. acetivoransi*-type model maintained/reduced the rTCA flux depending on the PYR influx, whereas the *T. takaii*-type model more significantly reduced it.

	Knockout of Pyruvate Carboxylase (**PYR–OAA**)	Knockout of Pyruvate synthase (**ACOA–PYR**)
T. takaii	The rTCA flux is impaired.	The rTCA flux is more significantly reduced.
D. acetivoransi	The rTCA flux is impaired.	The rTCA flux is maintained/reduced depending on the PYR influx.

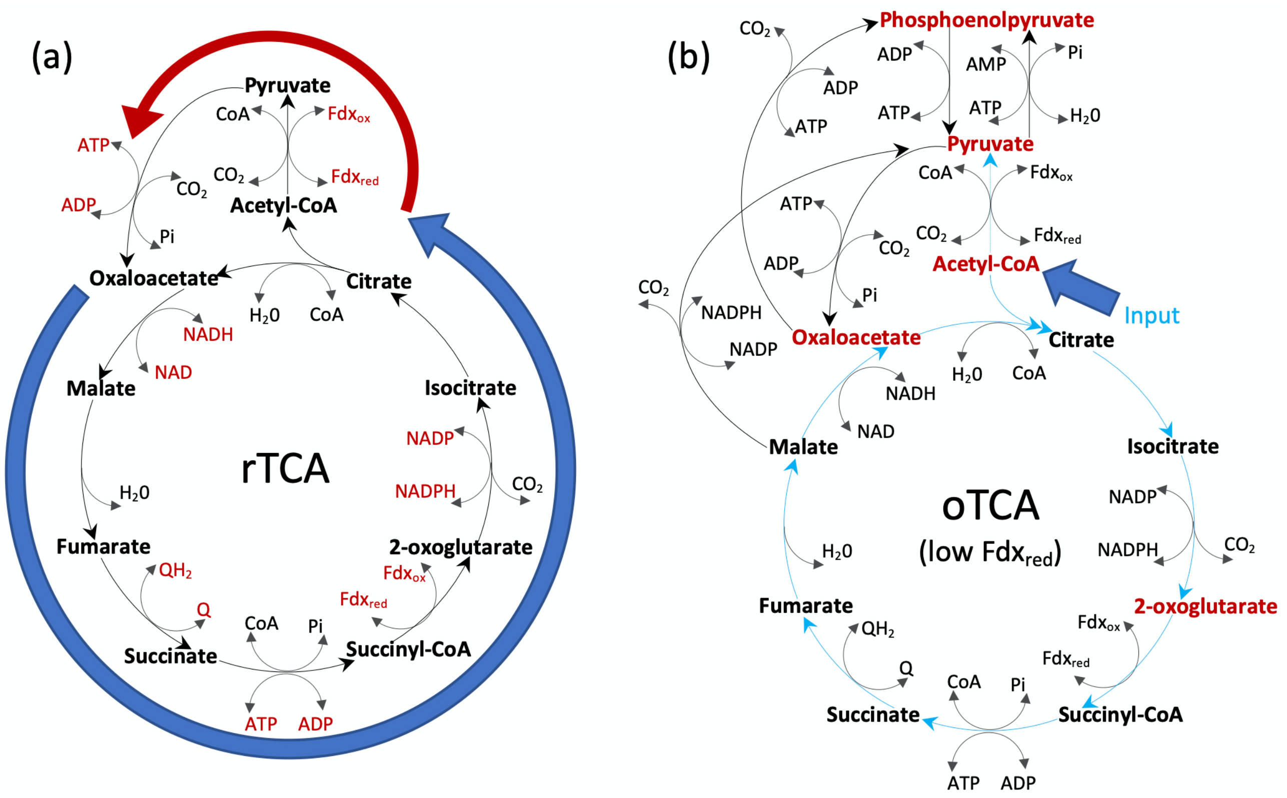

Figure S1. (a) The kinetic necessary condition for maintaining rTCA flux under autotrophic growth conditions with high concentrations of reduced ferredoxin which schematically illustrates the necessity of pyruvate carboxylase. (b) The kinetic network model under a heterotrophic growth condition with a low concentration of reduced ferredoxin reproduces oTCA fluxes in the presence of acetate. The directions of the fluxes shown in (b) were consistent with those observed for *D. acetivorans*i grown heterotrophically in the presence of acetate [19]. The light blue solid and broken lines in (b) respectively indicate fluxes changed by the acetyl-CoA influx and significantly reduced ones.

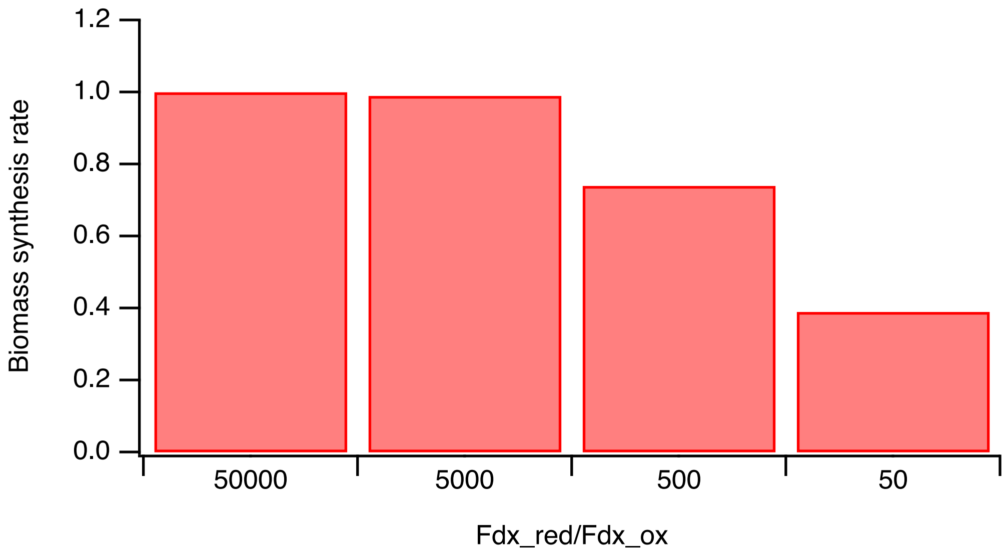

Fig S2. Oxidized ferredoxin (Fdx_{ox}) concentration dependence of chemolithoautotrophic biomass synthesis. The normal condition (the ratio of the reduced and oxidized ferredoxin, Fdx_red/Fdx_ox = 50000) was given by the ferredoxin concentrations Fdx_red = 2.5 x 10^{-3} mmol/ml and Fdx_ox = 5.0 x 10^{-8} mmol/ml. The other parameters listed in Table A1 were kept constant during increasing Fdx_ox. The biomass synthesis rate (Eq. B18) is normalized to that for the normal condition. The concentrations used for Fdx_ox are 5.0 x 10^{-8}, 5.0 x 10^{-7}, 5.0 x 10^{-6}, and 5.0 x 10^{-5} mmol/mL.

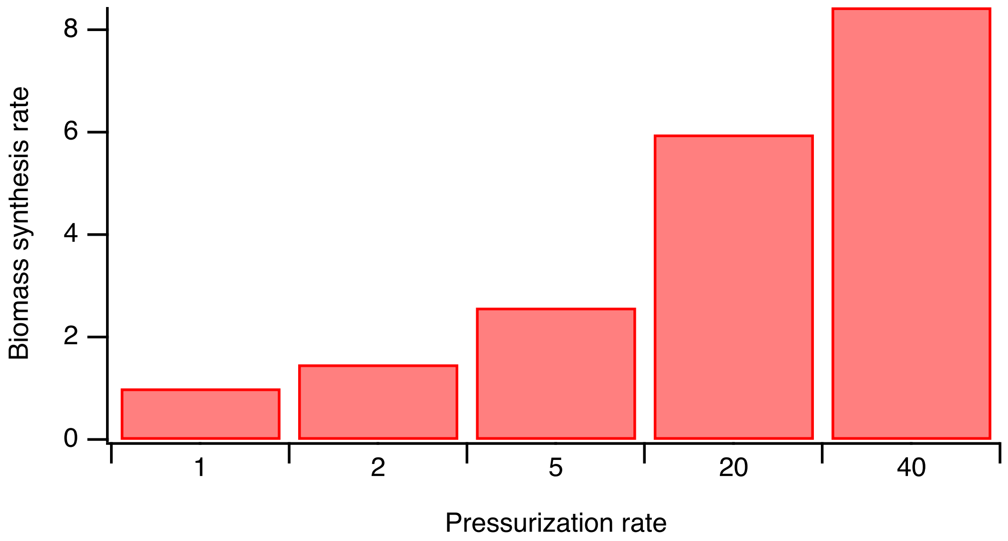

Fig S3. Biomass synthesis rate depending on the concentration of CO_2. The normal condition (the pressurization rate = 1) was assumed to be 0.0214 mmol/ml CO_2 [1,21]. The other parameters listed in Table A1 were kept constant during the pressurization of partial pressure of CO_2. The biomass synthesis rate (Eq. B18) is normalized to that for the normal condition. The CO_2 concentrations used are 0.0214, 0.0428, 0.107, 0.428, and 0.856 mmol/mL. The obtained result agrees with the growth rate observed for *D. acetivorans* [22].

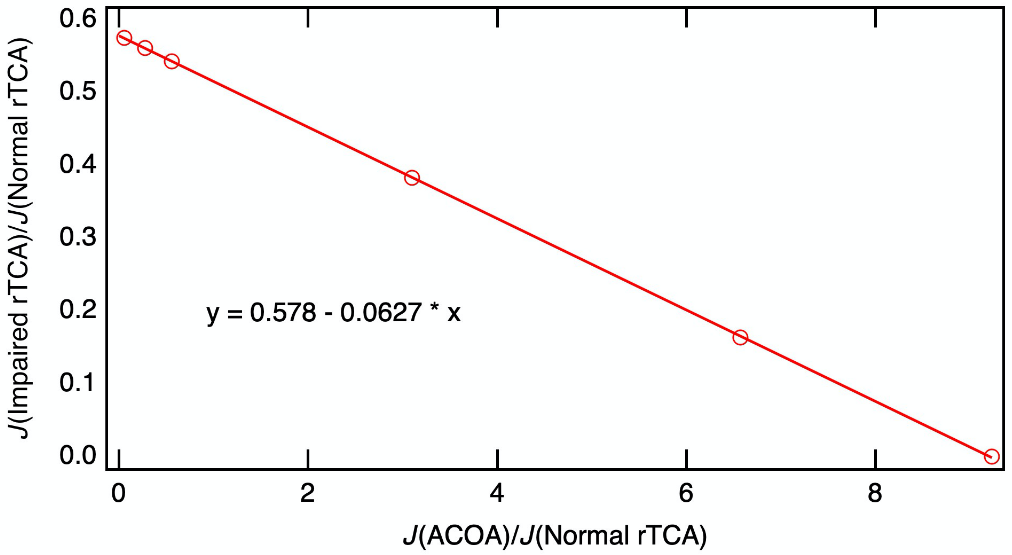

Fig S4. ACOA influx *J*(ACOA) dependence of ratio of the impaired reductive flux from AKG to ACOA and OAA *J*(Impaired rTCA) to the normal reductive flux from OAA to AKG *J*(Normal rTCA). The values less than 1 indicate *J*(Impaired rTCA) is reduced by *J*(ACOA), indicating the conflict between rTCA cycle and ACOA influx. The results shown in Fig. 1d corresponds to the result by the largest *J*(ACOA) shown here.

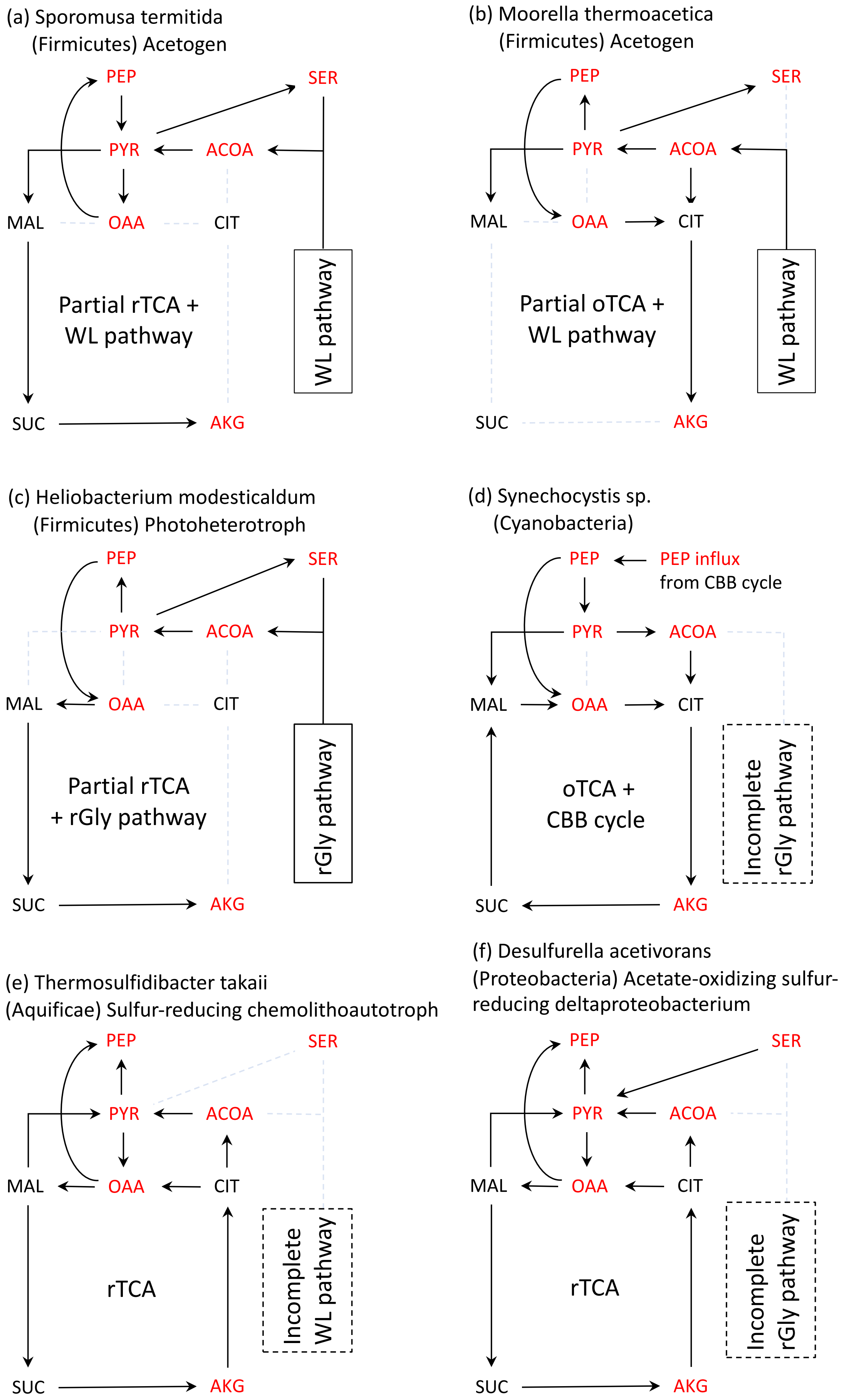
(a) Sporomusa termitida
(Firmicutes) Acetogen
PEP
SER
PYR
ACOA
MAL
OAA
CIT
Partial rTCA +
WL pathway
WL pathway
SUC
AKG
(b) Moorella thermoacetica
(Firmicutes) Acetogen
PEP
SER
PYR
ACOA
MAL
OAA
CIT
Partial oTCA +
WL pathway
WL pathway
SUC
AKG
(c) Heliobacterium modesticaldum
(Firmicutes) Photoheterotroph
PEP
SER
PYR
ACOA
MAL
OAA
CIT
Partial rTCA
+ rGly pathway
rGly pathway
SUC
AKG
(d) Synechocystis sp.
(Cyanobacteria)
PEP
PEP influx
from CBB cycle
PYR
ACOA
MAL
OAA
CIT
oTCA +
CBB cycle
Incomplete
rGly pathway
SUC
AKG
(e) Thermosulfidibacter takaii
(Aquificae) Sulfur-reducing chemolithoautotroph
PEP
SER
PYR
ACOA
MAL
OAA
CIT
rTCA
Incomplete
WL pathway
SUC
AKG
(f) Desulfurella acetivorans
(Proteobacteria) Acetate-oxidizing sulfur-
reducing deltaproteobacterium
PEP
SER
PYR
ACOA
MAL
OAA
CIT
rTCA
Incomplete
rGly pathway
SUC
AKG

(g) Desulfococcus multivorans
(Proteobacteria) Sulfate-reducing bacterium
with oxidative WL of ACOA

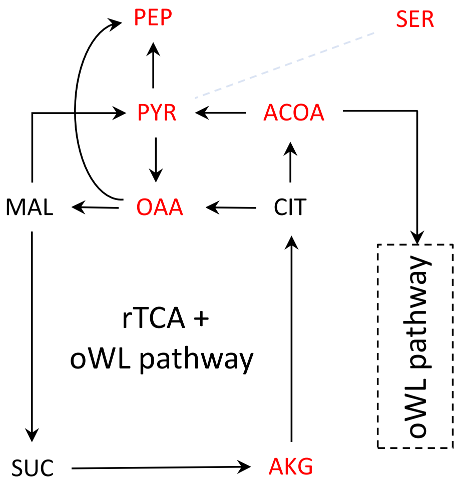

Figure S5. Schematic representation of carbon metabolic fluxes for deep-branching bacteria obtained from the kinetic network model simulation with enzymes identified by the KEGG genome database [23]. The light dashed lines without arrow indicate zero flux because of either totally or partially absence of enzymes. The "incomplete WL pathway" includes the cases where enzymes acting in the WL pathway do not exist at all, i.e., simply indicating that there is no ACOA influx. (a) *Sporomusa termitida* (Firmicutes), which is a H_2/CO_2-utilizing acetogen growing autotrophically and heterotrophically [24]. (b) *Moorella thermoacetica* (Firmicutes), which is an acetogenic anaerobic bacteria growing autotrophically and heterotrophically [25]. (c) *Heliobacterium modesticaldum* (Firmicutes), which is an anaerobic anoxygenic photoheterotroph [26,27]. It is suggested by the KEGG genome database that this organism possesses a reductive glycine (rGly) pathway [28] for carbon fixation. (d) *Synechocystis sp*. (Cyanobacteria), which possesses a Calvin–Benson–Bassham (CBB) cycle, thus grows photoautotrophically and photomixotrophically [29]. In contrast to (c), this organism lacks a few enzymes for rGly pathway. (e) *Thermosulfidibacter takaii* (Aquificae), which is a sulfur-reducing chemolithoautotroph and chemolithomixotroph [30]. (f) *Desulfurella acetivorans* (Proteobacteria), which is an acetate-oxidizing sulfur-reducing anaerobic deltaproteobacterium [19]. (g) *Desulfococcus multivorans* (Proteobacteria), which is a sulfate-reducing bacterium with oxidative WL pathway of ACOA [31]. The autotrophic growth condition with the high ratio Fdx_{red}/Fdx_{ox} (listed in Table A1) was assumed in organisms (a)–(g) grown autotrophically except for (d). In (d), a PEP influx yielded by the CBB cycle and a lower value of Fdx_{red}/Fdx_{ox} were assumed in the simulate for the oTCA cycle [32].

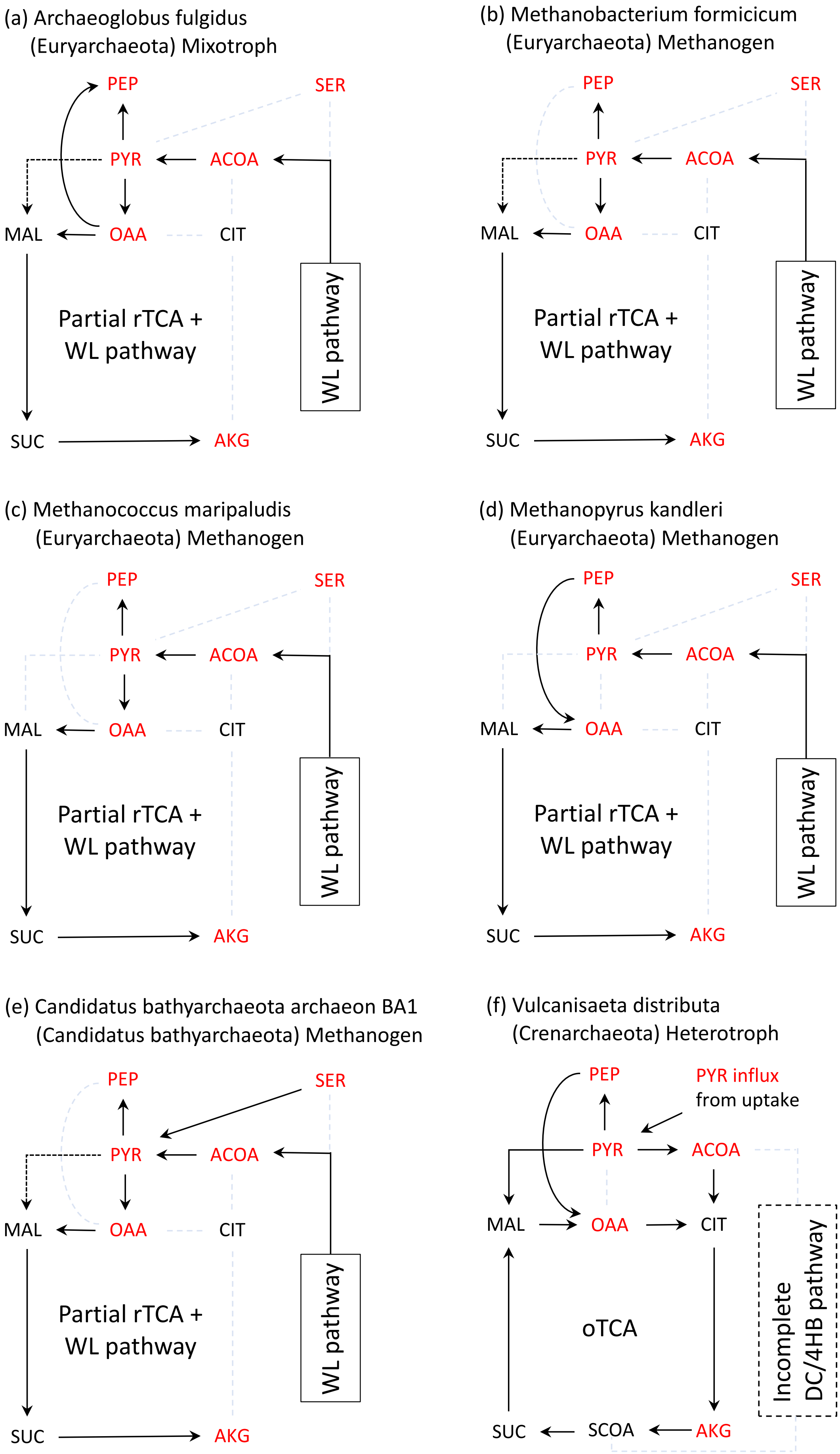
(a) Archaeoglobus fulgidus
(Euryarchaeota) Mixotroph
PEP
SER
PYR
ACOA
MAL
OAA
CIT
Partial rTCA +
WL pathway
WL pathway
SUC
AKG
(b) Methanobacterium formicicum
(Euryarchaeota) Methanogen
PEP
SER
PYR
ACOA
MAL
OAA
CIT
Partial rTCA +
WL pathway
WL pathway
SUC
AKG
(c) Methanococcus maripaludis
(Euryarchaeota) Methanogen
PEP
SER
PYR
ACOA
MAL
OAA
CIT
Partial rTCA +
WL pathway
WL pathway
SUC
AKG
(d) Methanopyrus kandleri
(Euryarchaeota) Methanogen
PEP
SER
PYR
ACOA
MAL
OAA
CIT
Partial rTCA +
WL pathway
WL pathway
SUC
AKG
(e) Candidatus bathyarchaeota archaeon BA1
(Candidatus bathyarchaeota) Methanogen
PEP
SER
PYR
ACOA
MAL
OAA
CIT
Partial rTCA +
WL pathway
WL pathway
SUC
AKG
(f) Vulcanisaeta distributa
(Crenarchaeota) Heterotroph
PEP
PYR influx
from uptake
PYR
ACOA
MAL
OAA
CIT
oTCA
Incomplete
DC/4HB pathway
SUC
SCOA
AKG

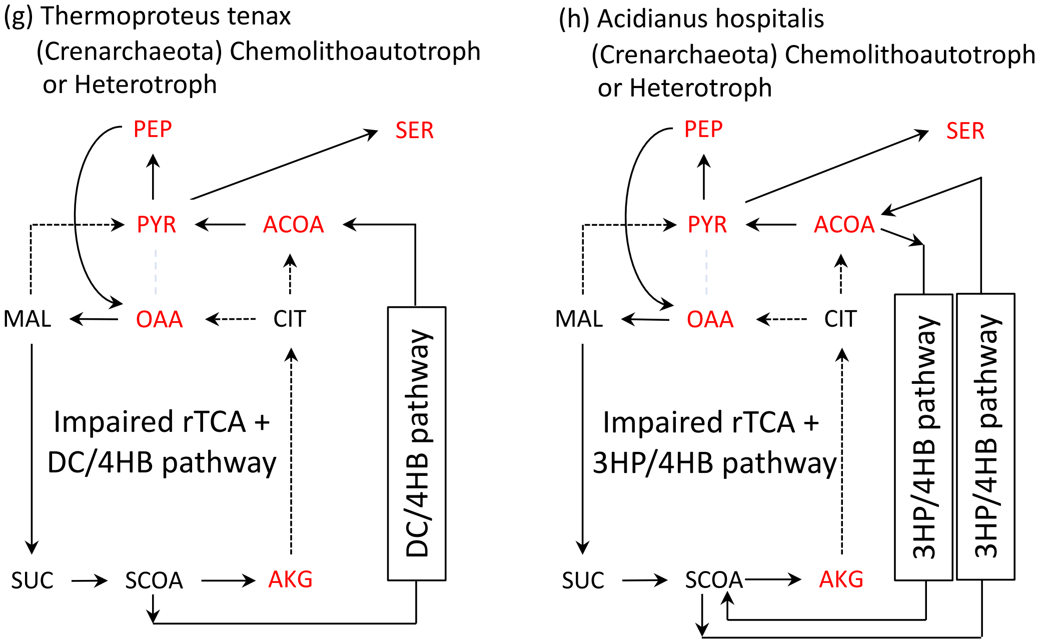

Figure S6. Schematic representation of carbon metabolic fluxes for deep-branching archaea obtained from the kinetic network model simulation with enzymes identified by the KEGG genome database [23]. The light dashed lines without arrow indicate zero flux because of either totally or partially absence of enzymes. Fluxes noticeably smaller than the others are indicated as dashed arrows. (a) *Archaeoglobus fulgidus* (Euryarchaeota), which grows heterotrophically or chemolithoautotrophically with sulfate or thiosulfate as electron acceptors [33]. (b) *Methanobacterium formicicum* (Euryarchaeota), which is a methanogenic archaea that is capable of producing CH_4 from the low carbon substrates such as MAL, PYR, methylamine, acetate, and CO_2 as a carbon source [34]. (c) *Methanococcus maripaludis* (Euryarchaeota), which is an autotrophic methanogen [35-37]. (d) *Methanopyrus kandleri* (Euryarchaeota), which is a H2-dependent autotrophic methanogen [38]. (e) *Candidatus Bathyarchaeota archaeon BA1* (Candidatus Bathyarchaeota), which is expected to be a methanogen [39]. (f) *Vulcanisaeta distributa* (Crenarchaeota), which is a heterotrophic anaerobic archaea [40]. This organism lacks a few enzymes for DC/4HB pathway. (g) *Thermoproteus tenax* (Crenarchaeota), which is a chemolithoautotrophy that possesses a dicarboxylate/4-hydroxybutyrate (DC/4HB) pathway [41]. (h) *Acidianus hospitalis* (Crenarchaeota), which is a chemolithoautotroph that possesses a 3-hydroxypropionate/4-hydroxybutyrate (3HP/4HB) pathway [42]. In (g) and (h), the rTCA fluxes are partially impaired by the ACOA influx from the DC/4HB and 3HP/4HB pathway, respectively, in the same manner as WL pathway. However, the inefficiency on the rTCA cycle should be acceptable if the DC/4HB and 3HP/4HB pathways more efficiently work for the carbon fixation. In the cases where complete WL pathway yielding ACOA influx into rTCA cycle (a–e), partial rTCA pathway commonly appears (see a–e). Most of those are seems to be autotrophic methanogen (b–e). The autotrophic growth condition with high Fdx_{red}/Fdx_{ox} (Table A1) was assumed in organisms (a)–(h) grown autotrophically except for (f). For (f) grown heterotrophically, a PYR influx from uptake and a lower value of Fdx_{red}/Fdx_{ox} were assumed in the simulate for the oTCA cycle. In (g) and (h), the DC/4HB

and 3HP/4HB pathways were modeled as a combination of additional ACOA influx and the conversion of succinyl-CoA (SCOA) to ACOA. Crenarchaeota in (g) and (h) grow heterotrophically [41,42], and the oTCA fluxes are simulated by lowering the ratio Fdx_{red}/Fdx_{ox}, though not shown in (g) and (h).

References

1. Wu, F., Yang, F., Vinnakota, K. C. & Beard, D. A. Computer modeling of mitochondrial tricarboxylic acid cycle, oxidative phosphorylation, metabolite transport, and electrophysiology. *J. Biol. Chem.* **282,** 24525–24537 (2007).
2. Li, X. *et al.* A database of thermodynamic quantities for the reactions of glycolysis and the tricarboxylic acid cycle. *J Phys Chem B* **114,** 16068–16082 (2010).
3. Alberty, R. A. Effect of temperature on standard transformed Gibbs energies of formation of reactants at specified pH and ionic strength and apparent equilibrium constants of biochemical reactions. *Journal of Physical Chemistry B* **105,** 7865–7870 (2001).
4. Alberty, R. A. Thermodynamic properties of weak acids involved in enzyme-catalyzed reactions. *J Phys Chem B* **110,** 5012–5016 (2006).
5. Li, X., Wu, F., Qi, F. & Beard, D. A. A database of thermodynamic properties of the reactions of glycolysis, the tricarboxylic acid cycle, and the pentose phosphate pathway. *Database (Oxford)* **2011,** bar005 (2011).
6. Beard, D. A., Vinnakota, K. C. & Wu, F. Detailed enzyme kinetics in terms of biochemical species: study of citrate synthase. *PLoS ONE* **3,** e1825 (2008).
7. Qi, F., Chen, X. & Beard, D. A. Detailed kinetics and regulation of mammalian NAD-linked isocitrate dehydrogenase. *Biochim Biophys Acta* **1784,** 1641–1651 (2008).
8. Bellei, M. *et al.* Control of reduction thermodynamics in [2Fe-2S] ferredoxins Entropy-enthalpy compensation and the influence of surface mutations. *J Inorg Biochem* **104,** 691–696 (2010).
9. Qi, F., Pradhan, R. K., Dash, R. K. & Beard, D. A. Detailed kinetics and regulation of mammalian 2-oxoglutarate dehydrogenase. *BMC Biochem* **12,** 53–15 (2011).
10. Cleland, W. W. The kinetics of enzyme-catalyzed reactions with two or more substrates or products. I. Nomenclature and rate equations. *Biochim Biophys Acta* **67,** 104–137 (1963).
11. Tronconi, M. A., Gerrard Wheeler, M. C., Maurino, V. G., Drincovich, M. F. & Andreo, C. S. NAD-malic enzymes of Arabidopsis thaliana display distinct kinetic mechanisms that support differences in physiological control. *Biochem. J.* **430,** 295–303 (2010).
12. Berman, K. M. & Cohn, M. Phosphoenolpyruvate synthetase of Escherichia coli. Purification, some properties, and the role of divalent metal ions. *J. Biol. Chem.* **245,** 5309–5318 (1970).
13. Wright, J. A. & Sanwal, B. D. Regulatory mechanisms involving nicotinamide adenine nucleotides as all teric effectors. II. Control of phosphoenolpyruvate carboxykinase. *J. Biol. Chem.* **244,** 1838–1845

(1969).

14. Giles, I. G., Poat, P. C. & Munday, K. A. The kinetics of rabbit muscle pyruvate kinase. Initial-velocity, substrate- and product-inhibition and isotopic-exchange studies of the reverse reaction. *Biochem. J.* **157,** 577–589 (1976).
15. Barden, R. E., Fung, C. H., Utter, M. F. & Scrutton, M. C. Pyruvate carboxylase from chicken liver. Steady state kinetic studies indicate a 'two-site' ping-pong mechanism. *J. Biol. Chem.* **247,** 1323–1333 (1972).
16. Millard, P., Smallbone, K. & Mendes, P. Metabolic regulation is sufficient for global and robust coordination of glucose uptake, catabolism, energy production and growth in Escherichia coli. *PLoS Comput Biol* **13,** e1005396 (2017).
17. Hoque, M. A., Ushiyama, H., Tomita, M. & Shimizu, K. Dynamic responses of the intracellular metabolite concentrations of the wild type and pykA mutant Escherichia coli against pulse addition of glucose or NH3 under those limiting continuous cultures. *Biochemical Engineering Journal* **26,** 38–49 (2005).
18. Peskov, K., Mogilevskaya, E. & Demin, O. Kinetic modelling of central carbon metabolism in Escherichia coli. *FEBS J* **279,** 3374–3385 (2012).
19. Mall, A. *et al.* Reversibility of citrate synthase allows autotrophic growth of a thermophilic bacterium. *Science* **359,** 563–567 (2018).
20. Sundararaj, S. *et al.* The CyberCell Database (CCDB): a comprehensive, self-updating, relational database to coordinate and facilitate in silico modeling of Escherichia coli. *Nucleic Acids Res.* **32,** D293–5 (2004).
21. Veech, R. L., Lawson, R., Cornell, N. W. & Krebs, H. A. Cytosolic Phosphorylation Potential. *J. Biol. Chem.* **254,** 6538–6547 (1979).
22. Steffens, L. *et al.* High CO2 levels drive the TCA cycle backwards towards autotrophy. *Nature* **592,** 784–788 (2021).
23. Kanehisa, M., Goto, S., Furumichi, M., Tanabe, M. & Hirakawa, M. KEGG for representation and analysis of molecular networks involving diseases and drugs. *Nucleic Acids Res.* **38,** D355–60 (2010).
24. Breznak, J. A., Switzer, J. M. & Seitz, H. J. Sporomusa-Termitida Sp-Nov, an H2/Co2-Utilizing Acetogen Isolated From Termites. *Arch. Microbiol.* **150,** 282–288 (1988).
25. Pierce, E. *et al.* The complete genome sequence of Moorella thermoacetica (f. Clostridium thermoaceticum). *Environ Microbiol* **10,** 2550–2573 (2008).
26. Kimble, L. K., Mandelco, L., Woese, C. R. & Madigan, M. T. Heliobacterium Modesticaldum, Sp-Nov, a Thermophilic Heliobacterium of Hot-Springs and Volcanic Soils. *Arch. Microbiol.* **163,** 259–267 (1995).
27. Sattley, W. M. *et al.* The genome of Heliobacterium modesticaldum, a phototrophic representative of the Firmicutes containing the simplest photosynthetic apparatus. *J Bacteriol* **190,** 4687–4696 (2008).

28. Sánchez-Andrea, I. *et al.* The reductive glycine pathway allows autotrophic growth of Desulfovibrio desulfuricans. *Nature Communications* **11,** 5090–12 (2020).
29. Zhang, S., Qian, X., Chang, S., Dismukes, G. C. & Bryant, D. A. Natural and Synthetic Variants of the Tricarboxylic Acid Cycle in Cyanobacteria: Introduction of the GABA Shunt into Synechococcus sp. PCC 7002. *Front Microbiol* **7,** 1972 (2016).
30. Nunoura, T. *et al.* A primordial and reversible TCA cycle in a facultatively chemolithoautotrophic thermophile. *Science* **359,** 559–563 (2018).
31. Dörries, M., Wöhlbrand, L., Kube, M., Reinhardt, R. & Rabus, R. Genome and catabolic subproteomes of the marine, nutritionally versatile, sulfate-reducing bacterium Desulfococcus multivorans DSM 2059. *BMC Genomics* **17,** 918–20 (2016).
32. Zhang, S. & Bryant, D. A. The Tricarboxylic Acid Cycle in Cyanobacteria. *Science* **334,** 1551– (2011).
33. Birkeland, N.-K., Schönheit, P., Poghosyan, L., Fiebig, A. & Klenk, H.-P. Complete genome sequence analysis of Archaeoglobus fulgidus strain 7324 (DSM 8774), a hyperthermophilic archaeal sulfate reducer from a North Sea oil field. *Stand Genomic Sci* **12,** 79–9 (2017).
34. Chellapandi, P., Bharathi, M., Sangavai, C. & Prathiviraj, R. Methanobacterium formicicum as a target rumen methanogen for the development of new methane mitigation interventions: A review. *Vet Anim Sci* **6,** 86–94 (2018).
35. Shieh, J. S. & Whitman, W. B. Pathway of acetate assimilation in autotrophic and heterotrophic methanococci. *J Bacteriol* **169,** 5327–5329 (1987).
36. Shieh, J. & Whitman, W. B. Autotrophic acetyl coenzyme A biosynthesis in Methanococcus maripaludis. *J Bacteriol* **170,** 3072–3079 (1988).
37. Ladapo, J. & Whitman, W. B. Method for isolation of auxotrophs in the methanogenic archaebacteria: role of the acetyl-CoA pathway of autotrophic CO2 fixation in Methanococcus maripaludis. *PNAS* **87,** 5598–5602 (1990).
38. Slesarev, A. I. *et al.* The complete genome of hyperthermophile Methanopyrus kandleri AV19 and monophyly of archaeal methanogens. *Proc. Natl. Acad. Sci. U.S.A.* **99,** 4644–4649 (2002).
39. Evans, P. N. *et al.* Methane metabolism in the archaeal phylum Bathyarchaeota revealed by genome-centric metagenomics. *Science* **350,** 434–438 (2015).
40. Mavromatis, K. *et al.* Complete genome sequence of Vulcanisaeta distributa type strain (IC-017). *Stand Genomic Sci* **3,** 117–125 (2010).
41. Siebers, B. *et al.* The complete genome sequence of Thermoproteus tenax: a physiologically versatile member of the Crenarchaeota. *PLoS ONE* **6,** e24222 (2011).
42. You, X.-Y. *et al.* Genomic analysis of Acidianus hospitalis W1 a host for studying crenarchaeal virus and plasmid life cycles. *Extremophiles* **15,** 487–497 (2011).
